# Efficient and selective photocatalytic CH_4_ conversion to CH_3_OH with O_2_ by controlling overoxidation on TiO_2_

**DOI:** 10.1038/s41467-021-24912-0

**Published:** 2021-08-02

**Authors:** Ningdong Feng, Huiwen Lin, Hui Song, Longxiao Yang, Daiming Tang, Feng Deng, Jinhua Ye

**Affiliations:** 1 State Key Laboratory of Magnetic Resonance and Atomic and Molecular Physics, National Center for Magnetic Resonance in Wuhan, CAS Key Laboratory of Magnetic Resonance in Biological Systems, Wuhan Institute of Physics and Mathematics, Innovation Academy for Precision Measurement Science and Technology, Chinese Academy of Sciences, Wuhan, China; 2 International Center for Materials Nanoarchitectonics (WPI-MANA), National Institute for Materials Science (NIMS), Ibaraki, Japan; 3 TJU-NIMS International Collaboration Laboratory, School of Material Science and Engineering, Tianjin University, Tianjin, China; 4 College of Materials Science and Technology, Jiangsu Key Laboratory of Electrochemical Energy Storage Technologies, Nanjing University of Aeronautics and Astronautics, Nanjing, China

**Keywords:** Catalytic mechanisms, Photocatalysis, Photocatalysis, Nanoscale materials

## Abstract

The conversion of photocatalytic methane into methanol in high yield with selectivity remains a huge challenge due to unavoidable overoxidation. Here, the photocatalytic oxidation of CH_4_ into CH_3_OH by O_2_ is carried out on Ag-decorated facet-dominated TiO_2_. The {001}-dominated TiO_2_ shows a durable CH_3_OH yield of 4.8 mmol g^−1^ h^−1^ and a selectivity of approximately 80%, which represent much higher values than those reported in recent studies and are better than those obtained for {101}-dominated TiO_2_. Operando Fourier transform infrared spectroscopy, electron spin resonance, and nuclear magnetic resonance techniques are used to comprehensively clarify the underlying mechanism. The straightforward generation of oxygen vacancies on {001} by photoinduced holes plays a key role in avoiding the formation of •CH_3_ and •OH, which are the main factors leading to overoxidation and are generally formed on the {101} facet. The generation of oxygen vacancies on {001} results in distinct intermediates and reaction pathways (oxygen vacancy → Ti–O_2_^•^ → Ti–OO–Ti and Ti–(OO) → Ti–O^•^ pairs), thus achieving high selectivity and yield for CH_4_ photooxidation into CH_3_OH.

## Abstract (web summary)

The photocatalytic conversion of CH_4_ into CH_3_OH with high activity and selectivity must avoid product overoxidation. Here, authors minimize overoxidation by using a (001)-dominated TiO_2_ nanosheet to circumvent CH_4_ overoxidation intermediates plus reaction pathways that occur on (101) facets.

## Introduction

Direct conversion of CH_4_ into methanol (CH_3_OH) is one of the most promising methods for methane optimization and utilization. However, CH_4_ is a very stable and inert molecule due to its negligible electron affinity, low polarizability, and high bonding energy for C–H (the first dissociation energy at 439 kJ mol^−1^). Thus, high temperatures and pressures are normally required to activate C–H bonds, which greatly increases capital investment and gives rise to operational risks and environmental problems^1^. Photocatalysis is a potential way to drive CH_4_ oxidation by utilizing photon energy instead of thermal energy. Upon excitation of semiconducting photocatalysts by photons, a series of highly active oxygen-containing radicals formed in photocatalytic CH_4_ oxidation can readily activate the C–H bond at room temperature^2–6^. However, the activation energy of the C–H bond in CH_4_ is much higher than that in the product (CH_3_OH)^7^. Thus, after the first C–H bond of CH_4_ is activated by active free radicals to form methyl or methoxy species, these species are easier to be activated and oxidized than CH_4_, eventually resulting in overoxidation of CH_3_OH to produce CO and CO_2_^8^. Especially for gas-phase CH_4_ oxidation, the product CH_3_OH can easily adsorb onto the surface of photocatalyst (such as TiO_2_ and ZnO loaded with various cocatalysts) and become overoxidized to CO and CO_2_^9–11^.

Due to unavoidable overoxidation, achieving high activity along with high selectivity at the same time is barely realized in the photocatalytic oxidation of CH_4_ to CH_3_OH^12–14^. The enhancement of one usually sacrifices the other. It has been found that in aqueous-phase CH_4_ oxidation, water can promote the desorption of products from active sites to avoid serious overoxidation^15,16^. Recently, Ma and Tang et al.^17^ reported direct photocatalysis of CH_4_ into CH_3_OH using a FeO_*x*_/TiO_2_ catalyst with H_2_O_2_ as the oxidant in an aqueous-phase system, resulting in a high CH_3_OH selectivity of ~90% but a relatively low yield of ~352 µmol g^−1^ h^−1^. However, the relatively high cost of H_2_O_2_ greatly limits its commercial application. Using O_2_ instead of H_2_O_2_ as the economically viable oxidant would represent substantial progress towards the oxidation of CH_4_^1^. In this case, Ye and coworkers^2^ used molecular O_2_ to photooxidize CH_4_ on Au/ZnO with a total organic compound selectivity of 95%. However, the CH_3_OH product tends to be overoxidized by •OH radicals formed in the reaction, which results in a CH_3_OH selectivity of less than 27% with a yield of 2.0 mmol g^−1^ h^−1^. More recently, Ye and coworkers used a CrO_*x*_-decorated Au/TiO_2_ photocatalyst to reduce the formation of •OH radicals in the reaction^18^, which increased the selectivity of CH_3_OH to ~50% with a yield of 2.5 mmol g^−1^ h^−1^. As such, the overoxidation of CH_3_OH should be partly attributed to the formation of •OH radicals. Furthermore, for the reported catalysts, CH_4_ can react with the photoinduced holes to form •CH_3_ on the surface, and the •CH_3_ can react with O_2_ and superoxide (O_2_^•^^−^) to mainly form CH_3_OOH rather than CH_3_OH^2,6,19^. It should be noted that CH_3_OOH can readily decompose into HCHO and H_2_O^2,9^. Therefore, as long as •CH_3_ and •OH exist in photocatalytic CH_4_ oxidation, it is difficult to improve the selectivity of CH_3_OH unless a new reaction pathway is introduced to reduce the formation of •CH_3_ and •OH through rational catalyst design. It has been reported that the intermediate photocatalytic species are closely related to the arrangement and coordination of the surface atoms on different crystal facets^20–24^.

In this work, two types of anatase TiO_2_, {001} or {101}-dominated TiO_2_ with Ag cocatalysts, are studied for CH_4_ photooxidation by O_2_. The {001}-dominated TiO_2_ shows a durable CH_3_OH yield of 4.8 mmol/g/h and a selectivity of ~80%, which are much higher values than those obtained on {101}-dominated TiO_2_. A comprehensive study by operando IR, ESR, and NMR reveals that the initial generation of oxygen vacancies on {001} by photoinduced holes can avoid the formation of •CH_3_ and •OH in the following reaction steps (Ti–O_2_^•^ → Ti–OO–Ti and Ti–(OO) → Ti–O^•^ pairs) to significantly reduce overoxidation. The scarcity of photogenerated oxygen vacancies on {101} leads to the formation of •CH_3_ and •OH, which is the main factor for overoxidation. This study provides a strategy to avoid overoxidation in reforming CH_4_ to CH_3_OH using other photocatalysts by controlling the generation of photogenerated oxygen vacancies.

## Results

### Selective photocatalytic oxidation of CH_4_ to CH_3_OH

**Fig. 1:**
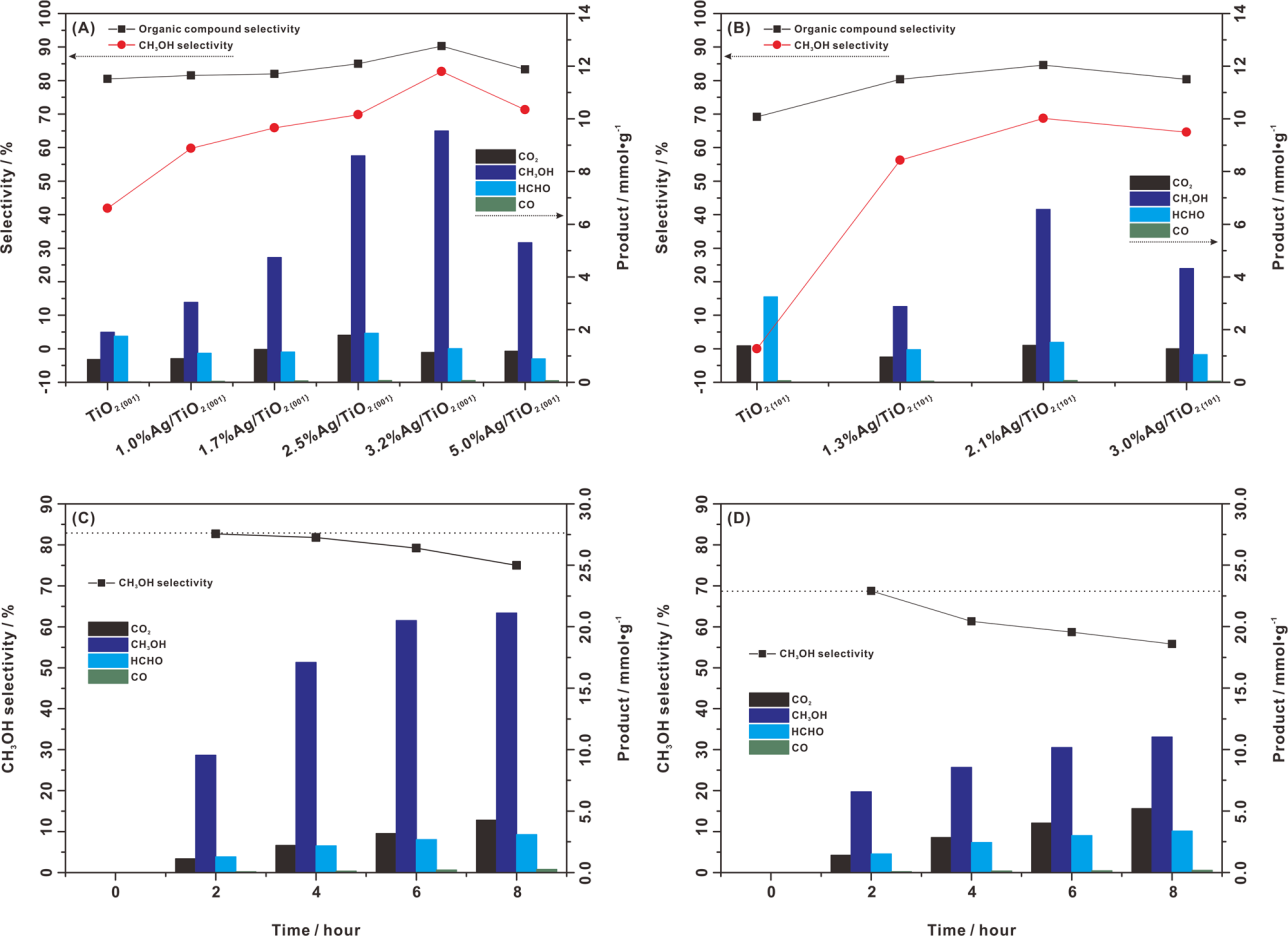
Photocatalytic CH_4_ conversion with molecular O_2_. CH_3_OH selectivity and product yields for a series of Ag-loaded TiO_2_ photocatalysts with predominantly exposed **A** {001} facets (0–5.0%Ag/TiO_2 {001}_) and **B** {101} facets (0–3.0%Ag/TiO_2 {101}_) under 2 h of irradiation. Time course for CH_3_OH selectivity and product yields for **C** 3.2%Ag/TiO_2 {001}_ and **D** 2.1%Ag/TiO_2 {101}_ under irradiation. Reaction conditions: 10 mg Ag/TiO_2_, 100 mL water, 2 MPa CH_4_, 0.1 MPa O_2_, 25 °C, light source: 300 W Xe lamp, light intensity of 450 mW/cm^2^.

Two types of anatase TiO_2_ (Supplementary Fig. S1) with predominantly exposed {001} or {101} facets were prepared, called TiO_2 {001}_ and TiO_2 {101}_, respectively. Silver (Ag) was chosen to decorate TiO_2_, which can trap photoinduced electrons to facilitate the separation and transfer of photoinduced carriers^25–27^. Typical oxide semiconductors, such as ZnO and TiO_2_, loaded with Ag cocatalysts have been demonstrated to exhibit good photocatalytic activity for CH_4_ activation^10,28^. Bare TiO_2_ (including TiO_2 {001}_ and TiO_2 {101}_) and that with variable Ag loading were evaluated for photocatalytic CH_4_ oxidation with molecular O_2_ as an oxidant (CH_4_:O_2_ ratio = 20:1). As shown in Fig. 1A, the products of CH_4_ photooxidation on TiO_2 {001}_ are CH_3_OH, HCHO, CO, and CO_2_. Among these products, the yield of CH_3_OH is 950 µmol g_cat._^−1^ h^−1^ and the selectivity of CH_3_OH is 42%. However, only deep oxidation products (HCHO, CO, and CO_2_) are formed on TiO_2 {101}_, and the production of CH_3_OH is invisible (Fig. 1B). This is consistent with previous reports of CH_4_ photooxidation by O_2_ over TiO_2_ (P25) and ZnO^2,9^. As such, the {001} facet should be favorable for the formation of CH_3_OH in CH_4_ photooxidation by O_2_, while the {101} facet is inclined to undergo deep oxidation.

We further studied the effect of the Ag loading amount on the catalytic performance. Clearly, a series of Ag loadings on TiO_2_ can effectively improve the activity of CH_4_ photooxidation. For TiO_2 {001}_, a 2.5%Ag and 3.2%Ag loading exhibited a high CH_3_OH yield of 4.3 mmol g_cat._^−1^ h^−1^ with a selectivity of ~70% and a yield of 4.8 mmol g_cat._^−1^ h^−1^ with an ultrahigh selectivity of ~80%, respectively (Fig. 1A and Table 1). For TiO_2 {101}_, the 2.1%Ag loading shows an optimal CH_3_OH yield of 3.3 mmol g_cat._^−1^ h^−1^ and a selectivity of ~68% (Fig. 1B and Table 1). Furthermore, with increasing irradiation time, the amounts of the total products (including CH_3_OH, HCHO, CO, and CO_2_) increase gradually with relatively stable CH_3_OH selectivity (74–80%) in CH_4_ photooxidation of 3.2%Ag/TiO_2 {001}_. (Fig. 1C and Table 1). Similar results can also be found for 2.5%Ag/TiO_2 {001}_ (Supplementary Fig. S2). The optimal CH_3_OH yield and selectivity on 3.2%Ag/TiO_2 {001}_ are much superior to those of most reported photocatalytic systems for CH_4_ oxidation to CH_3_OH (Supplementary Table S1). And we believe that this is the first study to achieve a high CH_3_OH yield and selectivity for CH_4_ photooxidation with O_2_ at room temperature. For 2.1%Ag/TiO_2 {101}_, although the amount of the deep oxidation product (HCHO, CO, and CO_2_) still increases with increasing irradiation time, the amount of CH_3_OH barely increases after 2 h of irradiation, which results in a significant decrease in CH_3_OH selectivity from 68% to 53% (Fig. 1D and Table 1). Therefore, it can be indicated that the mechanism for CH_4_ photooxidation by O_2_ on the {001} facet should be different from that on the {101} facet. The synergy between the cocatalyst Ag and the {001} facet can achieve selective and stable photocatalytic oxidation of CH_4_ into CH_3_OH by O_2_.

**Table 1:** Photocatalytic activity.

Photocatalysts	CH_4_ pressure (Mpa)	O_2_ pressure (Mpa)	Amount of product (μmol)				CH_3_OH yield (mmol/g/h)	CH_3_OH selectivity (%)	Organic compound selectivity (%)
			CH_3_OH	HCHO	CO	CO_2_			
TiO_2{001}_	2.0	0.1	19.0	17.5	0.2	8.6	0.95	41.9	80.5
1.0%Ag/TiO_2{001}_	2.0	0.1	30.4	11.0	0.4	9.0	1.52	59.8	81.5
1.7%Ag/TiO_2{001}_	2.0	0.1	47.4	11.5	0.5	12.5	2.37	65.9	82.0
2.5%Ag/TiO_2{001}_	2.0	0.1	86.0	18.7	0.7	17.8	4.30	69.8	85.0
3.2%Ag/TiO_2{001}_	2.0	0.1	95.4	12.8	0.7	11.3	4.77	79.4	90.0
5.0%Ag/TiO_2{001}_	2.0	0.1	53.0	8.9	0.6	11.8	2.65	71.3	83.3
TiO_2{101}_	2.0	0.1	0.0	26.3	0.5	11.2	0.00	0.0	69.2
1.3%Ag/TiO_2{101}_	2.0	0.1	28.7	12.4	0.4	9.6	1.44	56.2	80.4
2.1%Ag/TiO_2{101}_	2.0	0.1	65.6	15.1	0.7	14.0	3.28	68.7	84.6
3.0%Ag/TiO_2{101}_	2.0	0.1	43.1	10.5	0.4	12.7	2.15	64.6	80.4

Comparison of photocatalytic activity of TiO_2 {001}_ and TiO_2 {101}_ with and without a series of Ag loadings for the aqueous-phase photooxidation of CH_4_ by O_2_. Reaction conditions: 10 mg Ag/TiO_2_, 100 mL water, 25 °C reaction temperature, 2 h reaction time, light source: 300 W Xe lamp, light intensity of 450 mW cm^−2^.

### Characterization of Ag/TiO_2_ nanocatalysts

**Fig. 2:**
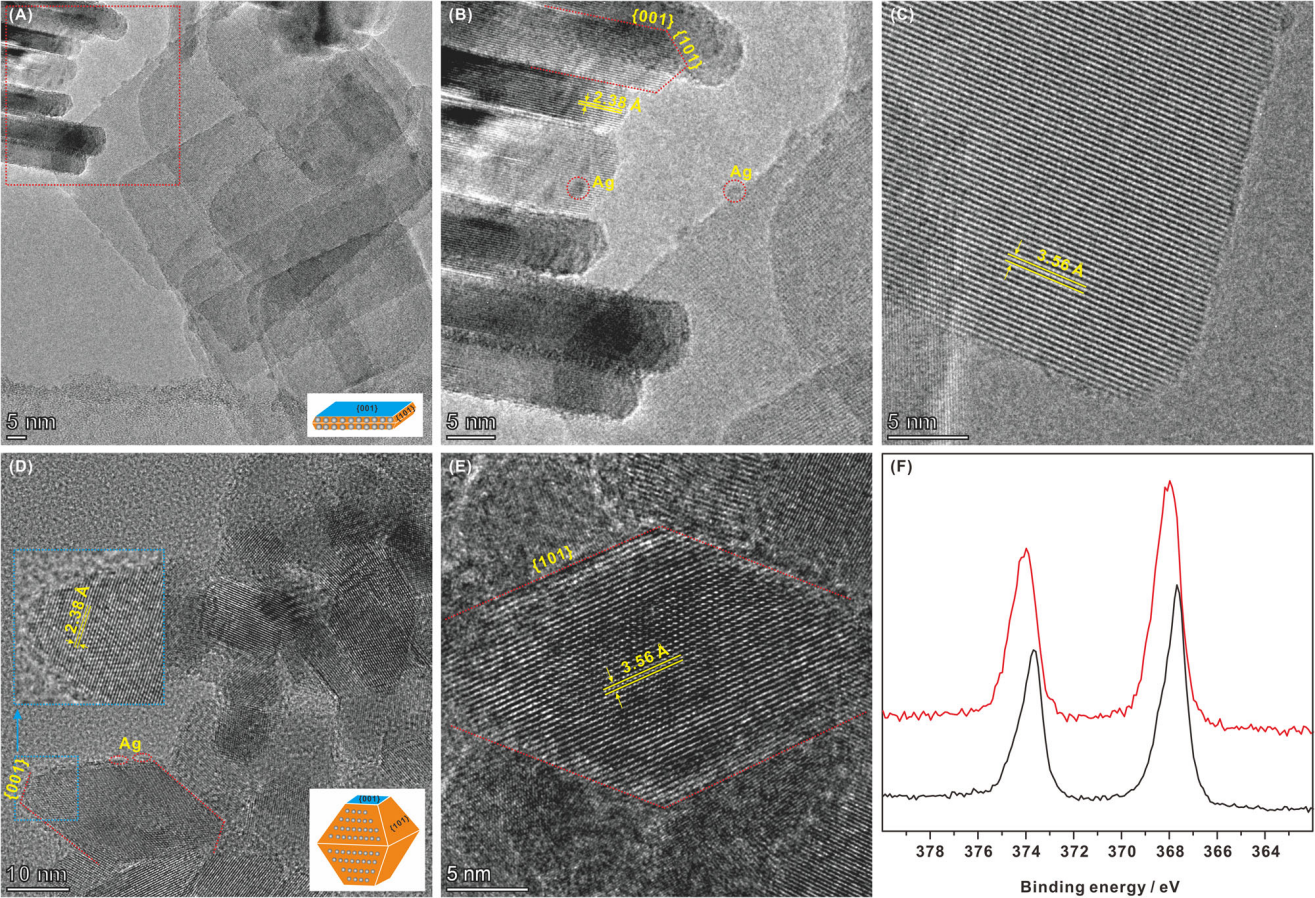
Photocatalyst characterization. **A**–**C** HRTEM images of 2.5%Ag/TiO_2 {001}_. **D**, **E** HRTEM images of 2.1%Ag/TiO_2 {101}_. **F** Ag 3*d* XPS spectra for 2.5%Ag/TiO_2 {001}_ (red line) and 2.1%Ag/TiO_2 {101}_ (black line).

To further clarify the correlation between photocatalytic performance and the crystal facet on Ag/TiO_2_ nanocatalysts, high-resolution transmission electron microscopy (HRTEM, 3100FEF spectrometer) and X-ray photoelectron spectroscopy (XPS) were performed to validate the morphology and elemental structure of 2.5%Ag/TiO_2 {001}_ and 2.1% Ag/TiO_2 {101}_. According to the HRTEM results, TiO_2 {001}_ exhibits a uniform square morphology with an average length of 54 nm and thickness of 4.6 nm (Fig. 2A), and TiO_2 {101}_ possesses an octahedral morphology with an average size of 17 nm (Fig. 2D). The lattice spacing parallel to the lateral facets and the top is 3.56 and 2.38 Å, corresponding to the {101} and {001} facets of anatase TiO_2_, respectively (Fig. 2B–E). As such, it can be found that both TiO_2 {001}_ and TiO_2 {101}_ nanoparticles coexpose {001} and {101} facets, and the percentage of dominant facets should be ~87% of the {001} facet in TiO_2 {001}_, and ~92% of the {101} facet in TiO_2 {101}_. Furthermore, HRTEM clearly shows that the Ag cocatalyst with a particle size of ~1.5 nm was selectively photodeposited onto the {101} facet of 2.5%Ag/TiO_2 {001}_ as shown in Fig. 2A, B. The chemical states for the Ag species on 2.5%Ag/TiO_2 {001}_ were characterized by XPS (Fig. 2F), and the Ag 3*d*_5/2_ peak observed at 368.0 eV can be associated with metal Ag nanoparticles. Similarly, on 2.1% Ag/TiO_2 {101}_, the Ag cocatalyst was also selectively photodeposited onto the {101} facet. However, due to the larger {101} facet, Ag can be dispersed better and smaller (<1.0 nm) than that on 2.5%Ag/TiO_2 {001}_ (Fig. 2D, E), which is reflected redshift observed for the Ag 3*d*_5/2_ peak (0.2 eV) in the XPS spectrum, as shown in Fig. 2F. According to the XPS results in Supplementary Fig. S3, titanium atoms exist in the form of Ti^4+^–O bonds in both TiO_2 {101}_ and TiO_2 {001}_^29^, as confirmed by the Ti 2*p*_3/2_ and 2*p*_1/2_ XPS peaks at 458.8 and 464.5 eV, and the O 1*s* XPS spectra show two peaks at 530.0 and 531.4 eV ascribed to lattice oxygen in Ti–O^2^^−^–Ti bonds and OH groups on the surface of TiO_2_^30^. In addition, no fluorine or chlorine atoms are present on Ag/TiO_2_ catalysts. The elemental composition of Ag and Ti was studied by energy dispersive spectroscopy (EDS), as shown in Supplementary Fig. S4. In addition, according to N_2_ adsorption and desorption experiments (Supplementary Fig. S5), the BET surface area of TiO_2 {101}_ (100.6 m^2^/g) is slightly higher than that of TiO_2 {001}_ (84.5 m^2^ g^−1^). Due to the particle accumulation, both TiO_2 {101}_ and TiO_2 {001}_ are porous with similar pore diameters 14.3 and 14.9 nm, respectively. As such, compared with TiO_2 {101}_, the higher photocatalytic activity and selectivity on TiO_2 {001}_ can be mainly ascribed to crystal plane and elemental structure rather than surface area and porosity.

### Reactive intermediate by operando and in situ characterization

**Fig. 3:**
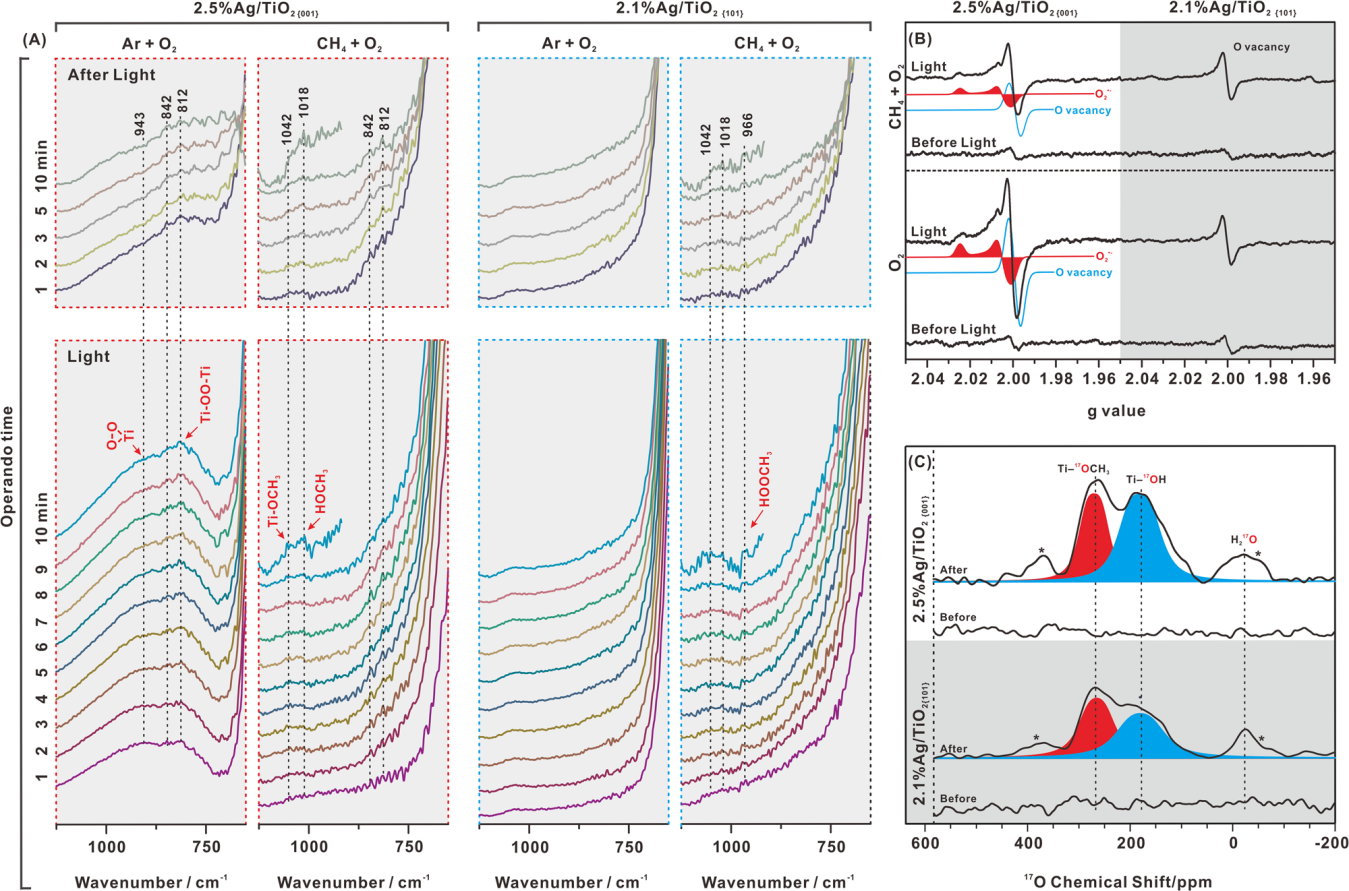
Reactive intermediates on the {001} and {101} facets. **A** Operando ATR-FTIR matrix for 2.5%Ag/TiO_2 {001}_ and 2.1% Ag/TiO_2 {101}_ with a 4 mL aqueous phase in a (Ar + O_2_) and (CH_4_ + O_2_) atmospheres before, during, and after light irradiation. **B** In situ ESR spectra for 2.5%Ag/TiO_2 {001}_ (left) and 2.1% Ag/TiO_2 {101}_ (right) with 300 µmol H_2_O loading in O_2_ (bottom) and (CH_4_ + O_2_) (upper) atmospheres before and during light irradiation. We fit the ESR data with the easyspin programs. **C** Ex situ ^17^O NMR spectra for 2.5%Ag/TiO_2 {001}_ (upper) and 2.1% Ag/TiO_2 {101}_ (lower) in a (CH_4_ + ^17^O_2_) atmosphere before and after light irradiation. The signals marked by asterisks are spinning sidebands. We fit the ^17^O NMR data with the Dimfit programs.

To gain insight into the different photocatalytic mechanisms for CH_4_ photooxidation on the {001} and {101} facets of TiO_2_, reactive intermediates were detected by operando ATR-FTIR experiments on 2.5%Ag/TiO_2 {001}_ and 2.1% Ag/TiO_2 {101}_ in the aqueous phase. The change in the difference spectra before light irradiation was also taken as the reference and indicator. It is certain at first that all the spectra observed under different conditions showed no change before light irradiation (Supplementary Figs. S6–S9). For the 2.5%Ag/TiO_2 {001}_ photocatalyst in the aqueous-phase saturated with Ar and O_2_ (Ar + O_2_) (Fig. 3A in 1st column), light irradiation led to the appearance of several peaks located at 943, 842, and 812 cm^−^^1^. The peaks in the 800–1000 cm^−^^1^ region were specifically assigned in previous reports^31,32^ to the O–O stretching of a surface peroxo species Ti–(OO) at 943 cm^−1^ ^32,33^, Ti–OOH at 842 cm^−1^ ^31^, and Ti–OO–Ti at 812 cm^−^^1^ ^32,34–36^. To validate this assignment, operando FTIR experiments were performed for the adsorption of H_2_O_2_ on the surface of TiO_2 {001}_ over time, and it was confirmed that the signal in the region of 800–1000 cm^−^^1^ corresponds to free and adsorbed H_2_O_2_ (Supplementary Fig. S10). The intensities of the peaks at 943 and 812 cm^−^^1^ increase immediately with the light irradiation time and drop down quickly after the light is turned off, suggesting that the intermediate species of Ti–(OO) and Ti–OO–Ti are photoinduced and metastable. Besides, these intermediate species show few changes with time under the light. However, when CH_4_ was introduced into the aqueous-phase system instead of Ar (CH_4_ + O_2_), both peaks at 943 and 812 cm^−^^1^ decreased significantly with light irradiation (Fig. 3A in 2nd column), suggesting the consumption of intermediate species (Ti–(OO) and Ti–OO–Ti) in the presence of CH_4_. Instead, two new peaks appear in the region of 1000–1100 cm^−^^1^, which can be assigned to the C–O stretching modes of CH_3_OH and Ti–O–CH_3_^37^.

Compared with the 2.5%Ag/TiO_2 {001}_ photocatalyst, no peak appears in the 800–1000 cm^−^^1^ region for the 2.1%Ag/TiO_2 {101}_ photocatalyst in the aqueous phase with Ar + O_2_ during light irradiation (Fig. 3A in 3rd column). It is suggested that the intermediates of Ti–(OO) and Ti–OO–Ti are not photoinduced on 2.1%Ag/TiO_2 {101}_. A similar change in spectral behavior is observed for 2.1%Ag/TiO_2 {101}_ in the aqueous phase with CH_4_ + O_2_ during light irradiation (Fig. 3A in 4th column), except for the presence of the peaks in the region of 1000–1100 cm^−^^1^ due to CH_3_OH and Ti–O–CH_3_ formation during light irradiation. Notably, the peaks in the region of 1000–1100 cm^−^^1^ on 2.1%Ag/TiO_2 {101}_ are much weaker than those on 2.5%Ag/TiO_2 {001}_ (Fig. 3A in 2nd and 4th columns) indicating that CH_4_ activation on the {001} facet should make it easier to form CH_3_OH and Ti–OCH_3_. In addition, a new peak located at 966 cm^−^^1^ can be assigned to the O–O stretching mode of CH_3_OOH (Fig. 3A in 4th column)^38^.

We used in situ ESR spectroscopy to follow the formation of paramagnetic intermediates on the {001} and {101} facets (Fig. 3B). For 2.5%Ag/TiO_2 {001}_ with 300 µmol H_2_O loading in a 24 µmol O_2_ atmosphere, two sets of ESR signals appeared upon light irradiation (Fig. 3B, left). The strong signal at *g* = 1.9996 is associated with oxygen vacancies arising from trapped photoinduced holes at surface oxygen. According to previous reports^39,40^, the signal for orthorhombic symmetry at *g*_*zz*_ = 2.023, *g*_*yy*_ = 2.007, and *g*_*xx*_ = 2.000 is ascribed to surface superoxide (Ti–O_2_^•^) sites. O_2_^•^^−^ is usually stabilized on a metallic cationic site so that the electrostatic interaction splits the 2π* antibonding orbitals by a certain amount (δ) due to the local cationic crystal field. The *g*_*zz*_ value can be measured by the equation *g*_*zz*_ = *g*_*e*_ + 2*λ*/*δ*, where *λ* is the spin–orbit coupling constant of oxygen^40^. As such, *g*_*zz*_ = 2.023 indicates that O_2_^•^^−^ is stabilized at the Ti sites for the oxygen vacancies. Combined with the operando FTIR results, the surface superoxide can be converted into surface peroxo species by the reduction of photoinduced electrons. However, when 30 µmol CH_4_ was introduced into the O_2_–Ag/TiO_2 {001}_ system ((CH_4_ + O_2_)–Ag/TiO_2 {001}_), both ESR signals decreased obviously upon light irradiation, indicating the consumption of paramagnetic intermediates (oxygen vacancies and Ti–O_2_^•^) in the presence of CH_4_. Compared with 2.5%Ag/TiO_2 {001}_, no surface superoxide appears for 2.1%Ag/TiO_2 {101}_ with a 300 µmol H_2_O loading in a 24 µmol O_2_ atmosphere during light irradiation (Fig. 3B, right), except for a small number of oxygen vacancies associated with the signal at *g* = 1.9996. Furthermore, the amount of oxygen vacancies does not change in the presence of 30 µmol CH_4_, indicating that oxygen vacancies are not involved in CH_4_ photooxidation by O_2_ on 2.1%Ag/TiO_2 {101}._ In addition, the pair of signals at *g* = 2.03 and 1.96 should be assigned to the background signal of in situ ESR tube (Supplementary Fig. S11).

Ex-situ ^17^O MAS NMR experiments were used to follow the transfer and evolution of oxygen in CH_4_ photooxidation by ^17^O_2_ on TiO_2_ photocatalysts (Fig. 3C). For 2.5%Ag/TiO_2 {001}_ in a 24 µmol ^17^O_2_ and 30 µmol CH_4_ atmosphere, three NMR signals appeared after 0.5 h of light irradiation. According to the previous reports^41^, the resonances at 480–570 ppm should arise from three coordinated oxygen species in the bulk of TiO_2_; the peaks at higher frequencies (600–750 ppm) can be assigned to two coordinated oxygen species on the surface of TiO_2_; the signals at much lower frequencies (100–300 ppm) can be attributed to hydroxyl groups (Ti–OH); the signals at −100–10 ppm can be attributed to adsorbed H_2_O. As such, the signals observed at −24 and 180 ppm are associated with H_2_^17^O and surface terminal hydroxyl (Ti–^17^OH), respectively (Fig. 3C, upper). Due to the deshielding effect of CH_3_, the ^17^O NMR signal of Ti–OCH_3_ should shift to a lower field than that of Ti–OH. Accordingly, we assign the ^17^O NMR signal at 263 ppm to Ti–OCH_3_ (Fig. 3C, upper), which is in good agreement with the formation of –OCH_3_ in the operando IR experiment for CH_4_ oxidation. Similar results are obtained on 2.1%Ag/TiO_2 {101}_ in a 24 µmol ^17^O_2_ and 30 µmol CH_4_ atmosphere before and after light irradiation (Fig. 3C, lower). However, the ^17^O signals at 180 ppm and 263 ppm are much lower than those for 2.5%Ag/TiO_2 {001}_, indicating that the O transfer efficiency from O_2_ to Ti–OCH_3_ and Ti–OH on the {001} facet in the photoreaction is much higher than that on the {101} facet. Combined with the operando FTIR and in situ ESR results, this can be attributed to the difference in the photocatalytic mechanism between the {001} and {101} facets, rather than the difference in the catalytic activity, because of the difference in reactivity (Fig. 1) is far from matching the difference in the O transfer efficiency.

### Distinct photocatalytic mechanism of CH_4_ oxidation by O_2_ on the {001} facet

**Fig. 4:**
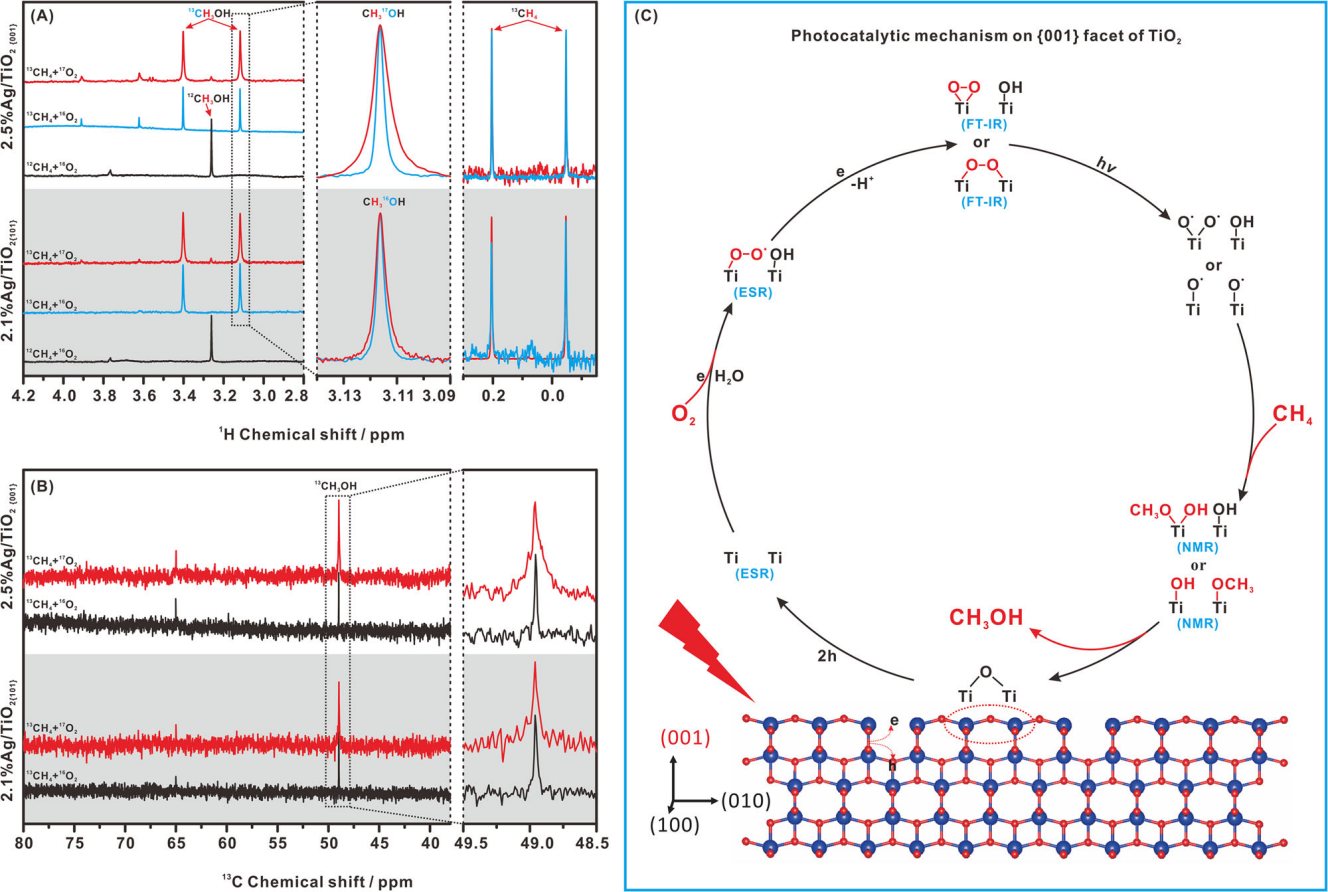
Photocatalytic mechanism. **A** ^1^H and **B** ^13^C NMR spectra for the product obtained from photocatalytic CH_4_ oxidation at a 4 h reaction time using 50 mL water, 80 kPa CH_4_, 20 kPa O_2_, and 10 mg Ag/TiO_2_. **C** Proposed photocatalytic mechanism for CH_4_ oxidation by O_2_ on the {001} facets of TiO_2_.

To detect the direct participation of CH_4_ and O_2_ in the formation of CH_3_OH, isotope labeling NMR experiments using ^13^CH_4_ and ^17^O_2_ were conducted under 80 kPa ^13^CH_4_ mixed with 20 kPa ^17^O_2_ on 2.5%Ag/TiO_2 {001}_ and 2.1% Ag/TiO_2 {101}_ in 50 mL water for 4 h. Figure 4A shows the ^1^H NMR spectra for the product obtained from photocatalytic CH_4_ oxidation in various atmospheres. For 2.5%Ag/TiO_2 {001}_ in a ^12^CH_4_ and ^16^O_2_ atmosphere, the strong peak at 3.26 ppm corresponds to ^12^CH_3_OH, and the weak peak at 3.77 ppm corresponds to trace ^12^CH_3_OOH. Using ^13^CH_4_ instead of ^12^CH_4_, both peaks split into two peaks due to ^1^H–^13^C J coupling (~140 Hz) for the methyl groups in the formed ^13^CH_3_OH (3.40 and 3.12 ppm) and ^13^CH_3_OOH (3.62 and 3.91 ppm) (Fig. 4A, upper). Similar results are also obtained on 2.1%Ag/TiO_2 {101}_ (Fig. 4A, lower). It can be indicated that the product CH_3_OH indeed originates from CH_4_ conversion on both 2.5%Ag/TiO_2 {001}_ and 2.1% Ag/TiO_2 {101}_. Interestingly, when the reaction was carried out on 2.5%Ag/TiO_2 {001}_ in a ^13^CH_4_ and ^17^O_2_ atmosphere, the FWHM (full width at half maximum) for the ^13^CH_3_OH signal obviously increased (Fig. 4A, upper). It should be noted that the FWHM of the signal for dissolved ^13^CH_4_ (0.20 and −0.04 ppm) in the ^13^CH_4_ and ^17^O_2_ atmosphere is consistent with that of dissolved ^13^CH_4_ in the ^13^CH_4_ and ^16^O_2_ atmosphere (Fig. 4A, upper). Thus, the widening of the ^13^CH_3_OH signal is due to weak J coupling (1.96 Hz) between the methyl proton and ^17^O, rather than any error related to the spectrometer and operation. On the other hand, when the reaction was carried out on 2.1% Ag/TiO_2 {101}_ in a ^13^CH_4_ and ^17^O_2_ atmosphere, the FWHM for the signal for the product ^13^CH_3_OH barely increased compared with that of the product ^13^CH_3_OH in a ^13^CH_4_ and ^16^O_2_ atmosphere (Fig. 4A, lower). Similar results were also found from the ^13^C NMR spectra (Fig. 4B). For Ag/TiO_2_ in the ^13^CH_4_ and ^16^O_2_ atmosphere, the two ^13^C NMR signals at 48.97 and 65.03 ppm can be attributed to ^13^CH_3_OH and ^13^CH_3_OOH, respectively, which further proves that the product CH_3_OH indeed originated from CH_4_ conversion on both 2.5%Ag/TiO_2 {001}_ and 2.1% Ag/TiO_2 {101}_. When the reaction was carried out on Ag/TiO_2_ in a ^13^CH_4_ and ^17^O_2_ atmosphere, the obvious peak broadening, originating from ^13^C–^17^O J coupling only occurred for the signal for ^13^CH_3_OH generated by 2.5%Ag/TiO_2 {001}_ (Fig. 4B, upper) but was barely observed for the signal for ^13^CH_3_OH generated by 2.1% Ag/TiO_2 {101}_ (Fig. 4B, lower). Combined with the results from ^1^H NMR experiments, it can be indicated that the oxygen in the CH_3_OH product mainly originates from the O_2_ in the photocatalytic CH_4_ oxidation on the {001} facet, while the oxygen in the CH_3_OH product should mainly originate from H_2_O and the surface oxygen of TiO_2_ in the photocatalytic CH_4_ oxidation on the {101} facet.

As in the previous reports^2,7,9,42^, the primary step of CH_4_ activation on typical oxide semiconductors should involve a reaction with surface O^−^ radical ions. When a TiO_2 {101}_ sample is illuminated under UV irradiation, the surface oxygen (Ti–O–Ti) captures one photoinduced hole to form Ti–O^•^^2–4^. As O^−^ is present on typical TiO_2_ and ZnO^2,9^, Ti–O^•^ can easily react with CH_4_ and H_2_O to form •CH_3_ and •OH:

1 $${{{{{\rm{Ti}}}}}}-{{{{{{\rm{O}}}}}}}^{\bullet }+{{{{{{\rm{CH}}}}}}}_{4}\to {{{{{\rm{Ti}}}}}}-{{{{{\rm{OH}}}}}}+\bullet {{{{{{\rm{CH}}}}}}}_{3},$$

2 $${{{{{\rm{Ti}}}}}}-{{{{{{\rm{O}}}}}}}^{\bullet }+{}^{-}{{{{{\rm{O}}}}}}{{{{{\rm{H}}}}}}\to {{{{{\rm{Ti}}}}}}-{{{{{\rm{OH}}}}}}+\bullet {{{{{\rm{OH}}}}}},$$

which was proven by ESR for 2.1% Ag/TiO_2 {101}_ in an aqueous solution (Supplementary Fig. S12). The active •CH_3_ can couple with the surface O^−^ and •OH to form Ti–OCH_3_ and CH_3_OH, as evidenced in the IR range of 1000–1100 cm^−^^1^ (Fig. 3A in 4th column). This is the reason why the oxygen in the CH_3_OH product mainly originates from H_2_O and Ti–O–Ti rather than O_2_ in the photocatalytic CH_4_ oxidation on 2.1% Ag/TiO_2 {101}_ (Fig. 4A, B). The O_2_ can be reduced by photoinduced electrons to form superoxide anion radicals (O_2_^•^^−^), which can easily react with •CH_3_ to form CH_3_OOH associated with the IR signal at 966 cm^−^^1^ (Fig. 3A in 4th column)^2,9,42^:

3 $${{{{{{\rm{O}}}}}}}_{2}+{{e}}\to {{{{{{\rm{O}}}}}}}_{2}^{\bullet -},$$

4 $$\bullet {{{{{{\rm{CH}}}}}}}_{3}+{{{{{{\rm{O}}}}}}}_{2}^{\bullet -}+{{{{{{\rm{H}}}}}}}^{+}\to {{{{{{\rm{CH}}}}}}}_{3}{{{{{\rm{OOH}}}}}}\bullet$$

CH_3_OOH can readily decompose into formaldehyde (HCHO) and H_2_O^2,9^, and HCHO and CH_3_OH can be overoxidized to CO_2_ and H_2_O by •OH radicals. Thus, as long as •CH_3_ and •OH radicals occur in photocatalytic CH_4_ oxidation, overoxidation should be unavoidable.

It has been found that the Ti–O–Ti bond angles of the {001} facets are more distorted than those of the {101} facets^21^, and there are much more pentacoordinated Ti^4+^ sites present on the {001} facets than that on the {101} facet^21,24^. All this leads to a higher surface energy (0.90 J m^−2^) on {001} facets than that (0.44 J m^−2^) on {101} facets^23^. In order to further prove the difference of the activity of oxygen sites and the formation of intermediates (oxygen (O) vacancy and surface peroxide species) on {001} and {101} facet of TiO_2_, the theoretical calculation has been performed (Supplementary Fig. S13). The energy (*E*_*f*_) required for the formation of O vacancy on {001} facet is much lower than that on the {101} facet, and the energy (*E*_ads_) released by O_2_ adsorption on O vacancy of {001} facet is much higher than that on O vacancy of {101} facet. In one word, the energy (2.717 eV) required for the formation of peroxide intermediate by O_2_ adsorption on {001} facet is much lower than that (3.983 eV) on {101} facet. Therefore, it can be concluded that, firstly, the O centers of the {001} facet are more active than those of the {101} facet, which is favorable for the formation of O vacancies on the {001} facet upon light irradiation, as confirmed by the ESR signal at *g* = 1.9996 in Fig. 3B; secondly, it should be easier to form peroxide intermediates by O_2_ adsorption on {001} facet, as confirmed by the operando FTIR experiment in Fig. 3A.

Thus, unlike CH_4_ activation on the {101} facet, the primary step of CH_4_ activation on the {001} facet should be the oxidation of surface oxygen by photoinduced holes to form oxygen vacancies:

5 $${{{{{\rm{Ti}}}}}}-{{{{{\rm{O}}}}}}-{{{{{\rm{Ti}}}}}}\,+\,2\,{{{{{\rm{h}}}}}}\,\to \,{{{{{\rm{Ti}}}}}}\,{{{{{\rm{Ti}}}}}},$$

which results in a distinct catalytic mechanism appearing on the {001} facet (Fig. 4C). The oxygen vacancy can stabilize the superoxide radical (O_2_^•^^−^), which should be formed by O_2_ reduction by photoinduced electrons, as in reaction 3, to form surface superoxide (Ti–O_2_^•^):

6 $${{{{{\rm{Ti}}}}}}\,{{{{{\rm{Ti}}}}}}+{{{{{{\rm{O}}}}}}}_{2}^{\bullet -}\to {{{{{\rm{Ti}}}}}}-{{{{{{\rm{OO}}}}}}}^{\bullet }\,{{{{{\rm{Ti}}}}}}$$

which is associated with the ESR signals at *g*_*zz*_ = 2.023, *g*_*yy*_ = 2.007, and *g*_*xx*_ = 2.000 (Fig. 3B). The surface superoxide can capture photoinduced electrons to form two types of surface peroxides (Ti–OO–Ti and Ti–(OO)):

7 $${{{{{\rm{Ti}}}}}}-{{{{{{\rm{OO}}}}}}}^{\bullet }\,{{{{{\rm{Ti}}}}}}\,+{{{{{\rm{e}}}}}}\,\to \,{{{{{\rm{Ti}}}}}}-{{{{{\rm{OO}}}}}}-{{{{{\rm{Ti}}}}}},$$

which corresponds to the appearance of O–O stretching bands at 812 and 943 cm^−^^1^ that show increased intensity with light irradiation time in the operando FTIR experiment, respectively (Fig. 3A in 1st column). With the presence of CH_4_, both Ti–OO–Ti and Ti–(OO) decrease significantly upon light irradiation (Fig. 3A in 2nd column). Theoretically, the dissociation barrier for the surface peroxides into Ti–O^•^ pairs on the {001} facet of anatase TiO_2_ is 1.0–1.4 eV, which can be overcome by the photon energy available from UV and visible light irradiation^43^:

8 $${{{{{\rm{Ti}}}}}}-{{{{{\rm{OO}}}}}}-{{{{{\rm{Ti}}}}}}\,+{{h}}{{{{{\rm{\nu }}}}}}\to {{{{{\rm{Ti}}}}}}-{{{{{{\rm{O}}}}}}}^{\bullet }\,{}^{\bullet }{{{{{\rm{O}}}}}}-{{{{{\rm{Ti}}}}}}.$$

The Ti–O^•^ pairs can split CH_4_ to generate adjacent surface methoxyl and hydroxyl groups (Ti–OCH_3_ HO–Ti), which are associated with the NMR signals observed 263 and 180 ppm, respectively (Fig. 3C), subsequently releasing CH_3_OH:

9 $${{{{{\rm{Ti}}}}}}-{{{{{{\rm{O}}}}}}}^{\bullet }\,{}^{\bullet }{{{{{\rm{O}}}}}}-{{{{{\rm{Ti}}}}}}+{{{{{{\rm{CH}}}}}}}_{4}\to {{{{{\rm{Ti}}}}}}-{{{{{{\rm{OCH}}}}}}}_{3}{{{{{\rm{HO}}}}}}-{{{{{\rm{Ti}}}}}}\to {{{{{\rm{Ti}}}}}}-{{{{{\rm{O}}}}}}-{{{{{\rm{Ti}}}}}}+{{{{{{\rm{CH}}}}}}}_{3}{{{{{\rm{OH}}}}}}.$$

This should be the reason why the oxygen in the CH_3_OH product mainly originates from O_2_ rather than H_2_O and Ti–O–Ti on 2.5% Ag/TiO_2 {001}_ (Fig. 4A, B), with almost no •CH_3_ and •OH generated in the photocatalytic CH_4_ oxidation on 2.5% Ag/TiO_2 {001}_ (Supplementary Fig. S12). Obviously, this reaction path can effectively hinder the formation of CH_3_OOH. According to the ESR spectra (Supplementary Fig. S12), there are no •OH species present in the photocatalytic CH_4_ oxidation on 2.5% Ag/TiO_2 {001}_, which can greatly reduce the overoxidation of CH_3_OH by •OH^2^. In addition, the presence of water in the reactions could promote the desorption of methoxyl/methanol from the surface of catalysts into an aqueous solution to avoid overoxidation of methanol to CO and CO_2_^15–17^, since the product CH_3_OH can physically or chemically adsorb onto the photocatalyst surface^4^, and can be further oxidized with the surface-active species (such as Ti–O^•^, peroxides, and superoxide) to form CO and CO_2_^9,10^. Thus, the aqueous phase is important to improve CH_3_OH selectivity in a photocatalytic CH_4_ oxidation reaction.

The proposed mechanism can further shed light on the results obtained for the photocatalytic activity and selectivity relative to the Ag loading amount on both TiO_2 {001}_ and TiO_2 {101}_. As observed in Fig. 1A, B, the initial increase in Ag loading can increase the photocatalytic activity and selectivity for both TiO_2 {001}_ and TiO_2 {101}_. To get insight into the role of Ag, the operando ATR-FTIR experiments on TiO_2 {001}_ and TiO_2 {101}_ have been performed (Supplementary Fig. S14). Similar to 2.5%Ag/ TiO_2 {001}_, light irradiation also led to two types of surface peroxo intermediates (Ti–OO–Ti and Ti–(OO)) for the TiO_2 {001}_ in the aqueous phase saturated with Ar and O_2_ (Ar + O_2_). On the other hand, similar to 2.1%Ag/TiO_2 {101}_, almost no peak appears in the 800–1000 cm^−^^1^ region for the TiO_2 {101}_ in the aqueous phase with Ar + O_2_ during light irradiation. It can be indicated that the formation of surface peroxo intermediates mainly occurred on the {001} facet of TiO_2_. The in situ ESR experiments were used to follow the formation of paramagnetic intermediates on TiO_2 {001}_ and TiO_2 {101}_ (Supplementary Fig. S15). Different from 2.5%Ag/ TiO_2 {001}_, no surface superoxide (Ti–O_2_^•^) was observed on both TiO_2 {001}_ and TiO_2 {101}_ in the presence of 300 μmol H_2_O and 24 μmol O_2_ during light irradiation, while a small number of oxygen vacancies was formed on TiO_2 {001}_. It can be indicated that Ag loading contributes to the formation of Ti–O_2_^•^ on TiO_2 {001}_. This may have two reasons: firstly, Ag as a cocatalyst can trap the photogenerated electrons to reduce molecular O_2_ to form O_2_^−^ species; secondly, the electron trapping on cocatalyst Ag can effectively improve the separation of photogenerated electrons and holes, which can lead to the increase of O vacancy formation arisen from the oxidation of surface oxygen by photogenerated hole on the {001} facet of TiO_2_ (Fig. 3B). As a result, the observable O_2_^−^ species is stabilized at the O vacancy on the {001} facet of TiO_2_ loaded with Ag (Fig. 3B). In addition to the role of Ag in separating the electron-hole pairs to increase the activity, more Ag loading can reduce the exposed area of the {101} facet since the Ag is mainly photodeposited on the {101} facet. Instead, the {001} facet will continue to be exposed to the solution, resulting in superior selectivity. However, excessive loading of Ag will decrease the activity because of the intrinsic light absorption by Ag, while the selectivity can remain high since the {001} facet can still be exposed to the solution.

## Discussion

In summary, we report the photocatalytic oxidation of CH_4_ into CH_3_OH by molecular O_2_ on anatase TiO_2_. To compare the microstructure and catalytic mechanism on the {001} and {101} facets of TiO_2_, two types of TiO_2_ with predominantly exposed {001} or {101} facets were prepared. By selectively photodepositing a Ag cocatalyst onto the {101} facet of TiO_2_ to facilitate the separation and transfer of photoinduced carriers, the CH_3_OH yield can be promoted significantly. According to studies based on operando FTIR, in situ ESR, and NMR techniques, completely different catalytic mechanisms exist for CH_4_ photooxidation by O_2_ on the {001} and {101} facets. It was found that oxygen vacancies on {001} facets are generated in a straightforward manner by photoinduced holes and that these photogenerated oxygen vacancies can stabilize superoxide radicals (Ti–O_2_^•^). Ti–O_2_^•^ can capture photoinduced electrons to form surface peroxides (Ti–OO–Ti and Ti–(OO)), and the surface peroxides dissociate into Ti–O^•^ pairs, which can split CH_4_ to release CH_3_OH directly. This distinct catalytic mechanism effectively avoids the formation of •CH_3_ and •OH, which are the main factors leading to overoxidation and are generally formed on the {101} facet. Thus, the optimized {001} facet-dominated TiO_2_ sample shows an impressively high CH_3_OH yield of 4.8 mmol g^−1^ h^−1^ with high CH_3_OH selectivity of ~80%. This study will provide a strategy to avoid overoxidation in CH_4_ reforming to CH_3_OH in other photocatalysts by controlling the generation of photoinduced oxygen vacancies.

## Methods

### Sample preparation

The anatase TiO_2_ with predominantly exposed {001} facets (TiO_2 {001}_) were prepared according to the previous report^44^. 25 mL of Ti(OBu)_4_ and 4 mL of hydrofluoric acid solution were mixed in a dried Teflon autoclave with a capacity of 100 mL, and then kept at 180 °C for 24 h. After being cooled to room temperature, the white powder was separated by high-speed centrifugation and washed with ethanol, 0.1 M NaOH aqueous solution, and deionized water several times to remove F^−^ on the surface.

The anatase TiO_2_ with predominantly exposed {101} facets (TiO_2 {101}_) were prepared according to the previous report^45^. Firstly, for the preparation of Ti(OH)_4_ precursor, 6.6 mL of TiCl_4_ was added to aqueous HCl (0.4 mol L^−1^) drop by drop under strong stirring in an ice bath to obtain an aqueous TiCl_4_. This TiCl_4_ aqueous was then added to aqueous NH_3_·H_2_O (5.5 wt.%) drop by drop under stirring. White Ti(OH)_4_ precipitate could be formed during the process. Afterward, the aqueous NH_3_·H_2_O (4.0 wt.%) was added to adjust the pH value to 6–7. After aging at room temperature for 2 h, the suspension was centrifuged, and the precipitate was washed with ethanol, 0.1 M NaOH aqueous solution, and deionized water several times to remove Cl^−^ on the surface.

4.0 g the fresh Ti(OH)_4_ precursor was first dispersed in the mixture of 30 mL deionized water and 30 ml isopropanol. After stirring and ultrasonic treatment, the suspension was transferred to a 100 mL Teflon-lined autoclave and heated for 24 h at 180 °C. The products were collected by centrifugation and washed with deionized ethanol one time and water three times. According to the EDX of TiO_2 {101}_ and TiO_2 {001}_ (Supplementary Fig. S16), there is no fluorine or chlorine atoms present on these TiO_2_.

The Ag-loaded TiO_2_ catalyst was prepared by in situ photodeposited reactions of AgNO_3_ with TiO_2_. Briefly, 0.4 g of the prepared TiO_2_ was suspended in an anaerobic aqueous solution containing deionized water (20.0 mL), CH_3_OH (5.0 mL), and a certain number of AgNO_3_. After 0.5 h irradiation under 300 W Xe lamp, the products were collected via centrifugation, then washed by water and dried at 60 °C.

### Characterization

A homemade spectral cell for attenuated total reflectance (ATR) Fourier transform infrared spectroscopy (FTIR) experiments is shown in Supplementary Fig. S17^32^. An internal reflection element (IRE) made of ZnSe (size: 50 mm × 20 mm × 3 mm, incident angle: 60°) was obtained from Pier Optics Co., Ltd. Japan. 20 mL TiO_2_ suspensions of 5 mg mL^−^^1^ in ethanol were drop casted on the IRE surface and dried in air. The IRE with TiO_2_ coating was then set into the homemade spectral cell. 4 mL of water was purged by the gas for different purposes and was then injected into the cell. Before the Operando ATR-FTIR experiments, the cell was kept in dark for 5 min.

An FTIR spectrometer (FT/IR-6300, Jasco Inc.) with liquid nitrogen cooled MCT detector was used and purged by dry nitrogen before ATR-FTIR experiments. The absorbance spectra ranged from 4000 to 500 cm^−^^1^ were obtained by repeated 32 scans with a resolution of 4 cm^−^^1^ and a processing time of 30 s. The background was monitored and recorded in every minute of 5 min before the light illumination. Thereafter, the spectra were obtained at every minute of 10 min with light illumination and after light illumination.

^17^O solid-state NMR spectra were acquired at 18.8 T on the Bruker Avance III spectrometers, equipped with a 4 mm double-resonance probe. The Larmor resonance frequencies for the ^1^H and ^17^O resonances were 800.4 and 108.5 MHz, respectively. ^17^O MAS NMR spectra were acquired using a typical π/2 pulse length of 2.5 μs, with a recycle delay of 1.0 s and a ^1^H decoupling field strength of 130 kHz. The experiments were carried out with a MAS frequency of 13.5 kHz. 120,000 scans were accumulated to acquire each spectrum. The chemical shifts of ^17^O resonance signals were referred to as liquid H_2_^17^O. Prior to ex-situ ^17^O NMR measurements, CH_4_ (30 μmol) and ^17^O_2_ (20μmol) were introduced into a glass ampule containing 0.05 g TiO_2_ catalyst under vacuum at the liquid N_2_ temperature, and then the glass ampule was sealed off. The photoreaction was performed in the sealed ampule under successive irradiation by a 300 W Xe lamp, and then the ampule was transferred into the rotor for the ex situ NMR measurements.

A homemade spectral cell for in situ electron spin resonance (ESR) experiments is shown in Supplementary Fig. S18. In situ ESR experiments were carried at X-band using a JOEL FA 2000 spectrometer. The microwave frequency was 9.1 GHz, the modulation amplitude was 0.1 mT, the microwave power was 5 mW and the experimental temperature was 25 °C. The g values of the radical species were referenced to Mn-marker. The Mn-marker is Mn^2+^ in the CaO with *g* = 2.0009.

### Photocatalytic measurements

The photocatalytic methane oxidation reaction tests were conducted in a 230 mL batch reactor equipped with a quartz window to allow light irradiation. 10 mg catalyst was dispersed in 100 mL water by ultrasonication for 10 min. Then, the mixture was added into a glass cell with a volume of 30 mL, and the glass cell was placed in the batch reactor. The actual working volume decreased to 100 mL. The batch reactor was purged with O_2_ (purity, 99.99995%) for 15 min to exhaust air. After that, the reactor vessel was pressurized with 0.1 MPa O_2_ and 2 MPa CH_4_ (purity, 99.9995%). Subsequently, the reactor was loaded into a cold-water bath, and the solution was stirred at 1200 rpm. 300 W Xe lamp was used as the light source with wavelength ranging from 300 to 500 nm and the light intensity of 450 mW cm^−2^. A thermocouple was inserted into the solution to directly detect the temperature of the liquid solution. During the reaction process, the temperature of the liquid solution was maintained at 25 °C. After the reaction, the reactor was cooled in an ice bath to a temperature below 10 °C. Then the gas product was collected, and the concentrations of gas products were analyzed by gas chromatograph (GC, Shimadzu) equipped with methanizer and flame ionization detector. The liquid phase of the reaction mixture product was collected by centrifugation. The liquid product was analyzed by nuclear magnetic resonance spectroscopy (NMR) and the colorimetric method.

## Supplementary information

Efficient and Selective Photocatalytic CH4 Conversion to CH3OH with O2 by Controlling Overoxidation on TiO2

## Data Availability

The data that support the plots within this paper and other findings of this study are available from the corresponding authors upon reasonable request.

## References

[1] Ravi M, Ranocchiari M, van Bokhoven JA (2017). The direct catalytic oxidation of methane to methanol—a critical assessment. Angew. Chem. Int. Ed..

[2] Song H (2019). Direct and selective photocatalytic oxidation of CH_4_ to oxygenates with O_2_ on cocatalysts/ZnO at room temperature in water. J. Am. Chem. Soc..

[3] Liu F (2017). Transfer channel of photoinduced holes on a TiO_2_ surface as revealed by solid-state nuclear magnetic resonance and electron spin resonance spectroscopy. J. Am. Chem. Soc..

[4] Yang L (2020). Surface water loading on titanium dioxide modulates photocatalytic water splitting. Cell Rep. Phys. Sci..

[5] Volodin AM, Cherkashin AE (1982). ERS spectrum of methyl radicals on ZnO surface. React. Kinet. Catal. Lett..

[6] Maldotti A, Molinari A, Amadelli R (2002). Photocatalysis with organized systems for the oxofunctionalization of hydrocarbons by O_2_. Chem. Rev..

[7] Latimer AA (2017). Understanding trends in C–H bond activation in heterogeneous catalysis. Nat. Mater..

[8] Song H (2019). Solar-energy-mediated methane conversion. Joule.

[9] Chen X (2016). Photocatalytic oxidation of methane over silver decorated zinc oxide nanocatalysts. Nat. Commun..

[10] Yu X, De Waele V, Löfberg A, Ordomsky V, Khodakov AY (2019). Selective photocatalytic conversion of methane into carbon monoxide over zinc-heteropolyacid-titania nanocomposites. Nat. Commun..

[11] Li Z, Pan X, Yi Z (2019). Photocatalytic oxidation of methane over CuO-decorated ZnO nanocatalysts. J. Mater. Chem. A.

[12] Murcia-López S, Villa K, Andreu T, Morante JR (2014). Partial oxidation of methane to methanol using bismuth-based photocatalysts. ACS Catal..

[13] Villa K, Murcia-López S, Andreu T, Morante JR (2015). Mesoporous WO_3_ photocatalyst for the partial oxidation of methane to methanol using electron scavengers. Appl. Catal. B.

[14] Murcia-López S (2017). Controlled photocatalytic oxidation of methane to methanol through surface modification of beta zeolites. ACS Catal..

[15] Latimer AA, Kakekhani A, Kulkarni AR, Nørskov JK (2018). Direct methane to methanol: the selectivity–conversion Limit and design strategies. ACS Catal..

[16] Lustemberg PG (2018). Direct conversion of methane to methanol on Ni-Ceria surfaces: metal–support interactions and water-enabled catalytic conversion by site blocking. J. Am. Chem. Soc..

[17] Xie J (2018). Highly selective oxidation of methane to methanol at ambient conditions by titanium dioxide-supported iron species. Nat. Catal..

[18] Song H (2020). Selective photo-oxidation of methane to methanol with oxygen over dual-cocatalyst-modified titanium dioxide. ACS Catal..

[19] Agarwal N (2017). Aqueous Au-Pd colloids catalyze selective CH_4_ oxidation to CH_3_OH with O_2_ under mild conditions. Science.

[20] Tian N, Zhou Z-Y, Sun S-G, Ding Y, Wang ZL (2007). Synthesis of tetrahexahedral platinum nanocrystals with high-index facets and high electro-oxidation activity. Science.

[21] Selloni A (2008). Anatase shows its reactive side. Nat. Mater..

[22] Yin Y, Alivisatos AP (2005). Colloidal nanocrystal synthesis and the organic–inorganic interface. Nature.

[23] Yang HG (2008). Anatase TiO_2_ single crystals with a large percentage of reactive facets. Nature.

[24] Pan J, Liu G, Lu GQ, Cheng H-M (2011). On the true photoreactivity order of {001}, {010}, and {101} facets of anatase TiO_2_ crystals. Angew. Chem. Int. Ed..

[25] Feng N (2013). Understanding the high photocatalytic activity of (B, Ag)-codoped TiO_2_ under solar-light irradiation with XPS, solid-state NMR, and DFT calculations. J. Am. Chem. Soc..

[26] Zhu X (2020). Facet-selective deposition of a silver–manganese dual cocatalyst on potassium hexatitanate photocatalyst for highly selective reduction of carbon dioxide by water. Appl. Catal. B.

[27] Gao D (2020). Core-shell Ag@Ni cocatalyst on the TiO_2_ photocatalyst: one-step photoinduced deposition and its improved H_2_ evolution activity. Appl. Catal. B.

[28] Yu X (2020). Stoichiometric methane conversion to ethane using photochemical looping at ambient temperature. Nat. Energy.

[29] Wang Z (2013). Visible-light photocatalytic, solar thermal and photoelectrochemical properties of aluminium-reduced black titania. Energy Environ. Sci..

[30] Wang Y, Feng C, Zhang M, Yang J, Zhang Z (2010). Enhanced visible light photocatalytic activity of N-doped TiO_2_ in relation to single-electron-trapped oxygen vacancy and doped-nitrogen. Appl. Catal. B.

[31] Nakamura R, Nakato Y (2004). Primary intermediates of oxygen photoevolution reaction on TiO_2_ (rutile) particles, revealed by in situ FTIR absorption and photoluminescence measurements. J. Am. Chem. Soc..

[32] Nakamura R, Imanishi A, Murakoshi K, Nakato Y (2003). In situ FTIR studies of primary intermediates of photocatalytic reactions on nanocrystalline TiO_2_ films in contact with aqueous solutions. J. Am. Chem. Soc..

[33] Ding Q (2020). Unravelling the water oxidation mechanism on NaTaO_3_-based photocatalysts. J. Mater. Chem. A.

[34] Yamada H, Hurst JK (2000). Resonance Raman, optical spectroscopic, and EPR characterization of the higher oxidation states of the water oxidation catalyst, cis,cis- (bpy)Ru(OH)_2_O^4+^. J. Am. Chem. Soc..

[35] Root, D. E., Mahroof-Tahir, M., Karlin, K. D. & Solomon, E. I. Effect of protonation on peroxo-copper bonding: spectroscopic and electronic structure study of Cu-2((UN-O-)(OOH)^(2+)^. *Inorg. Chem.* **37**, 4838–4848 (1998). 10.1021/ic980606c 11670647

[36] Liu HF, Frei H (2020). Observation of O-O bond forming step of molecular Co_4_O_4_ cubane catalyst for water oxidation by rapid-scan FT-IR spectroscopy. ACS Catal..

[37] Chiarello GL, Ferri D, Selli E (2018). In situ attenuated total reflection infrared spectroscopy study of the photocatalytic steam reforming of methanol on Pt/TiO_2_. Appl. Surf. Sci..

[38] Chen Z, Wang C (2006). Rate constants of the gas-phase reactions of CH_3_OOH with O_3_ and NO_x_ at 293K. Chem. Phys. Lett..

[39] Attwood AL, Murphy DM, Edwards JL, Egerton TA, Harrison RW (2003). An EPR study of thermally and photochemically generated oxygen radicals on hydrated and dehydrated titania surfaces. Res. Chem. Intermed..

[40] Che, M. & Tench, A. J. *Advances in Catalysis* (eds Eley, D. D., Pines, H., & Weisz, P. B.) Vol. 32, 1–148 (1983).

[41] Li Y (2017). Distinguishing faceted oxide nanocrystals with ^17^O solid-state NMR spectroscopy. Nat. Commun..

[42] Ito T, Lunsford JH (1985). Synthesis of ethylene and ethane by partial oxidation of methane over lithium-doped magnesium oxide. Nature.

[43] Liu L, Wang Z, Pan C, Xiao W, Cho K (2013). Effect of hydrogen on O_2_ adsorption and dissociation on a TiO_2_ anatase (001) surface. ChemPhysChem.

[44] Han X, Kuang Q, Jin M, Xie Z, Zheng L (2009). Synthesis of titania nanosheets with a high percentage of exposed (001) facets and related photocatalytic properties. J. Am. Chem. Soc..

[45] Liu L (2013). Anion-assisted synthesis of TiO_2_ nanocrystals with tunable crystal forms and crystal facets and their photocatalytic redox activities in organic reactions. J. Phys. Chem. C.

