# Supplementary material for: Efficient and selective photocatalytic CH4 conversion to CH3OH with O2 by controlling overoxidation on TiO2
Source: Nat Commun. 2021 Aug 2;12:4652. doi: 10.1038/s41467-021-24912-0 (PMC8329221; doi:10.1038/s41467-021-24912-0)
Supplement: Supplementary file 1 — Efficient and Selective Photocatalytic CH4 Conversion to CH3OH with O2 by Controlling Overoxidation on TiO2 [file 41467_2021_24912_MOESM1_ESM.pdf]

Supplementary information

Efficient and Selective Photocatalytic CH_4 Conversion to CH_3OH with O_2 by Controlling Overoxidation on TiO_2

Ningdong Feng*[1,2,5], Huiwen Lin[2,4,5], Hui Song[2,5], Longxiao Yang[1], Daiming Tang[2], Feng Deng[1], Jinhua Ye* [2,3]

[1] State Key Laboratory of Magnetic Resonance and Atomic and Molecular Physics, National Center for Magnetic Resonance in Wuhan, CAS Key Laboratory of Magnetic Resonance in Biological Systems, Wuhan Institute of Physics and Mathematics, Innovation Academy for Precision Measurement Science and Technology, Chinese Academy of Sciences, Wuhan 430071, China.

[2] International Center for Materials Nanoarchitectonics (WPI-MANA), National Institute for Materials Science (NIMS), 1-1 Namiki, Tsukuba, Ibaraki 305-0044, Japan.

[3] TJU-NIMS International Collaboration Laboratory, School of Material Science and Engineering, Tianjin University, Tianjin, 300072, P. R. China.

[4] College of Materials Science and Technology, Jiangsu Key Laboratory of Electrochemical Energy Storage Technologies, Nanjing University of Aeronautics and Astronautics, Nanjing 210016, China.

[5] These authors contributed equally to this work.

*E-mail: ningdong.feng@wipm.ac.cn, Jinhua.YE@nims.go.jp.

Table S1. Comparison of catalytic activity in photooxidation of methane to methanol.

Entry	Catalyst	Oxidant	Reaction condition	CH_3OH		CH_4 conversion (%)	Ref.
				Amount (μmol)	Selectivity (%)		
1	3.2%Ag/TiO_2 {001}	O_2	10 mg catalyst, 2.0 MPa CH_4 (2000 mL), 25 ºC, 4 h, 300 W Xe lamp,	173	79	0.31	This work
2	0.33 wt% FeO_x/TiO_2	H_2O_2	10 mg catalyst, 70 μmol CH_4, 8 μmol H_2O_2, 25 ºC, 3h, 300 W Xe lamp	10.56	90	14.9	[1]
3	Au-CoO_x/TiO_2	O_2	10 mg catalyst, 2.0 MPa CH_4 (2000 mL), 0.1 MPa O_2, 25 ºC, 4 h, 300 W Xe lamp	36	45	0.1	[2]
4	0.1 wt% Pd/ZnO	O_2	10 mg catalyst, 2.0 MPa CH_4 (2000 mL), 0.1 MPa bar O_2, 25 ºC, 4 h 300 W Xe lamp	108.2	36	0.35	[3]
5	$BiVO_4$	H_2O	300 mg catalyst, 20% CH_4/He, 55 ºC, 2 h, 450 W immersion medium-pressure Hg lamp with UVC-visible radiation	12.48	51	-	[4]
6	WO_3 mesoporous	H_2O + $FeCl_3$ (2 mM)	300 mg catalyst, 20% CH_4/He, 55 ºC, 2 h, 450 W immersion medium-pressure Hg lamp with UVC-visible radiation	33.3	37.4	0.3%	[5]
7	La-doped WO_3 mesoporous	H_2O	300 mg catalyst, 20% CH_4/He, 55 ºC, 2 h, 450 W immersion medium-pressure Hg lamp with UVC-visible radiation	18.84	47	0.17	[6]

Note: the CH_4 conversion in ref. 4 cannot be calculated because the amount of CH_4 was not provided.

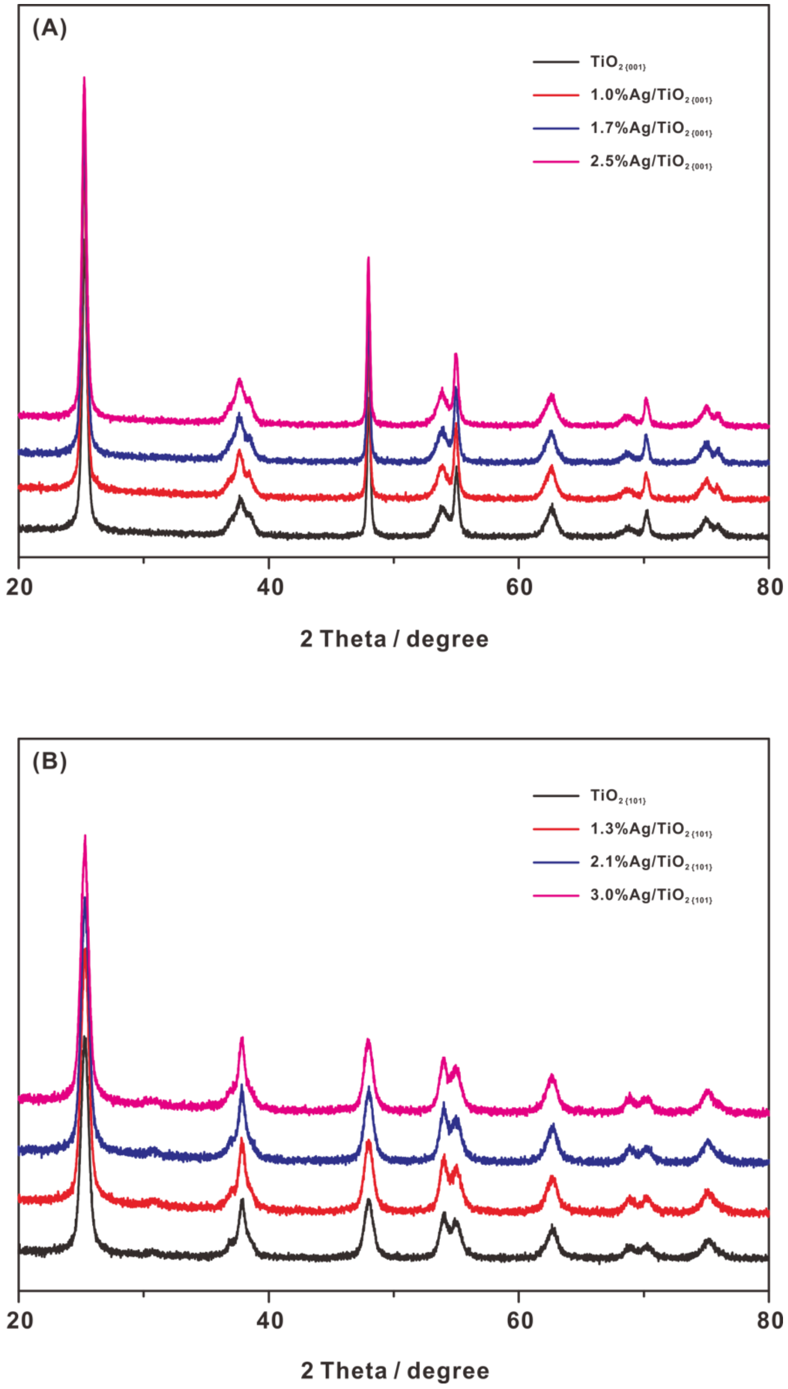

Figure S1. XRD spectra of (A) $TiO_{2\ \{001\}}$ loaded with variable Ag (0 ~ 2.5%) and (B) $TiO_{2\ \{001\}}$ loaded with variable Ag (0 ~ 3.0%).

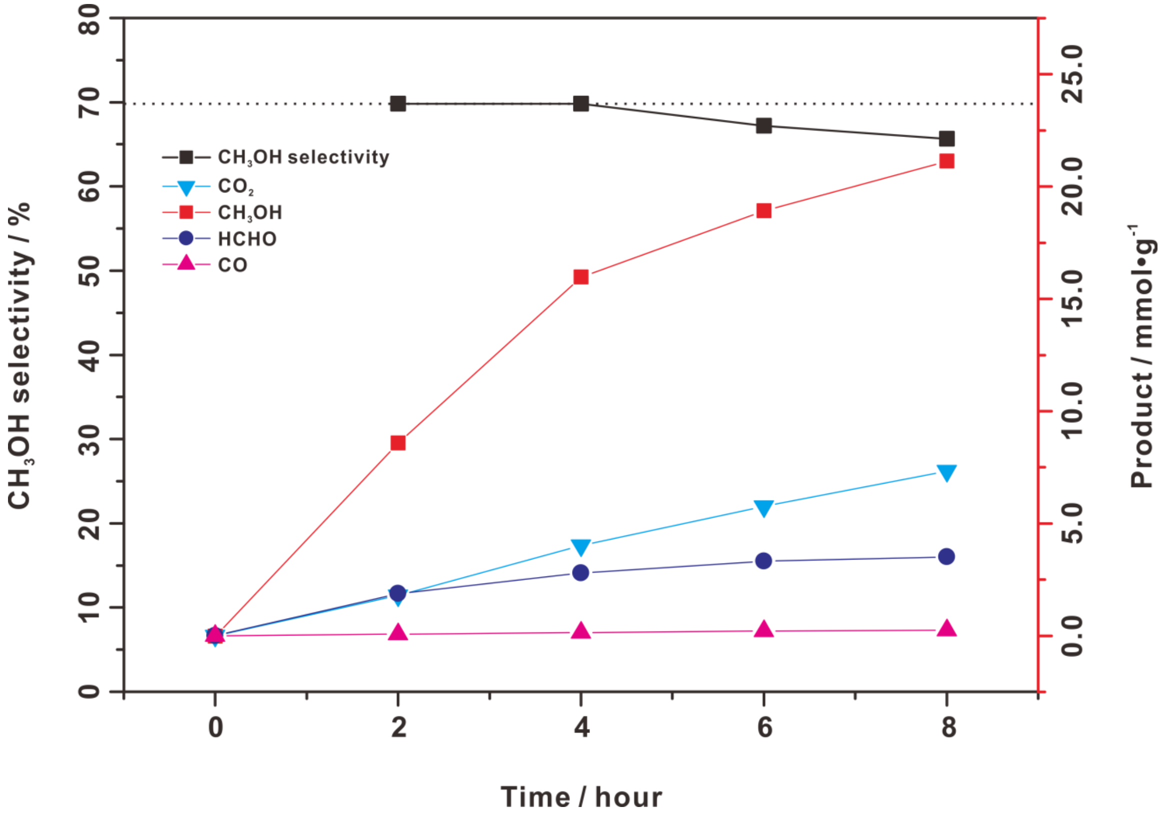

Figure S2. Time course of CH_3OH selectivity and product yields for 2.5%Ag/$TiO_{2\,\{001\}}$ under irradiation. Reaction conditions: 10 mg Ag/TiO_2, 100 mL water, 2 MPa CH_4, 0.1 MPa O_2, 25 °C, light source: 300 W Xe lamp, light intensity 100 mW/cm^2.

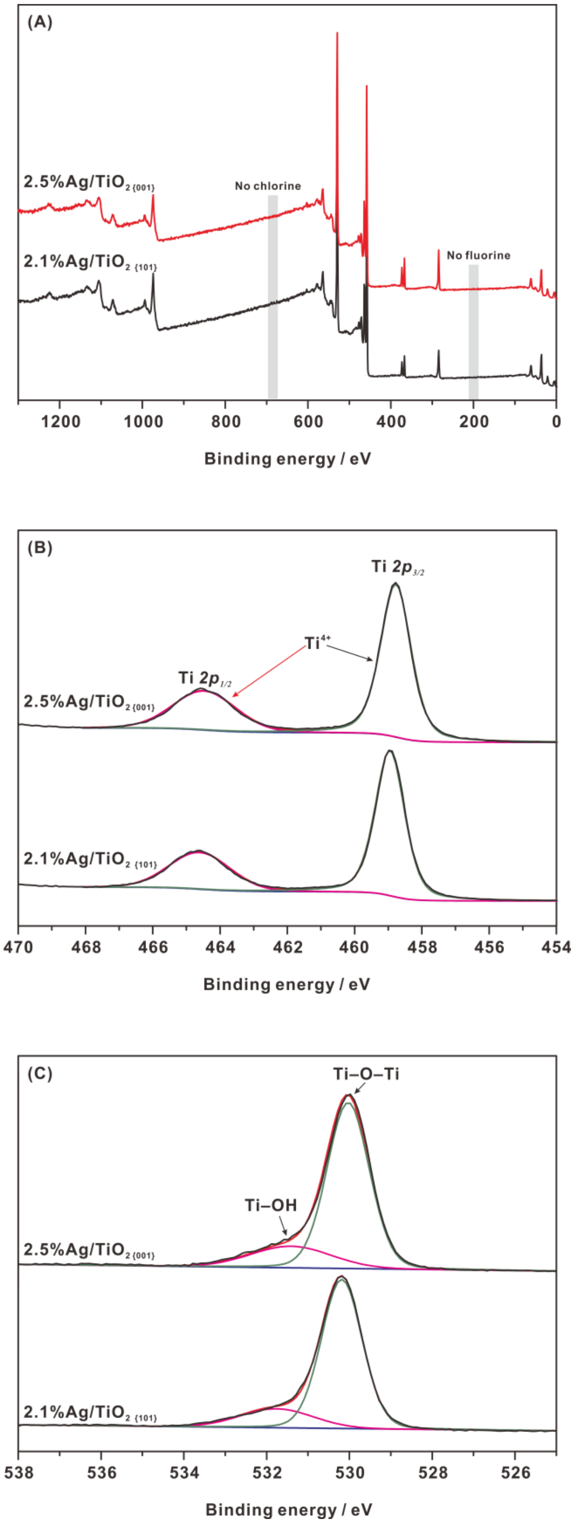

Figure S3. (A) Wide scan, (B) Ti *2p*, and (C) O *1s* XPS spectra of 2.5%Ag/$TiO_{2\ \{001\}}$ and 2.1%Ag/$TiO_{2\ \{101\}}$.

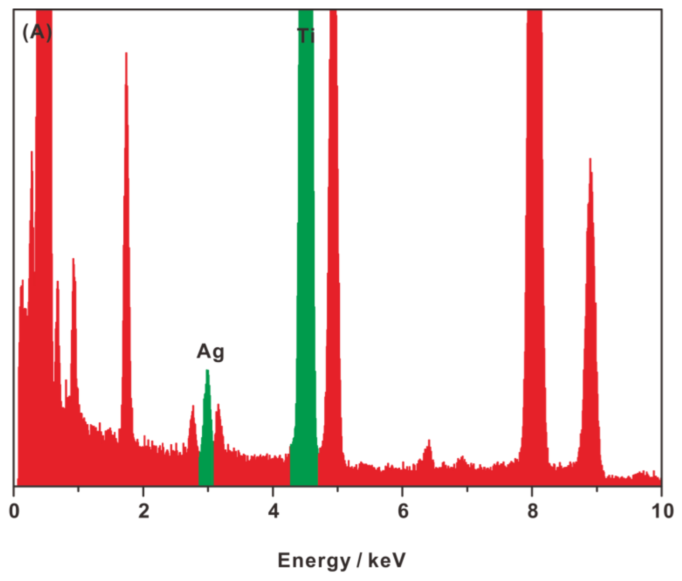

Element	Weight %	Atomic %
Ag L	5.51%	2.52%
Ti K	94.49%	97.48%

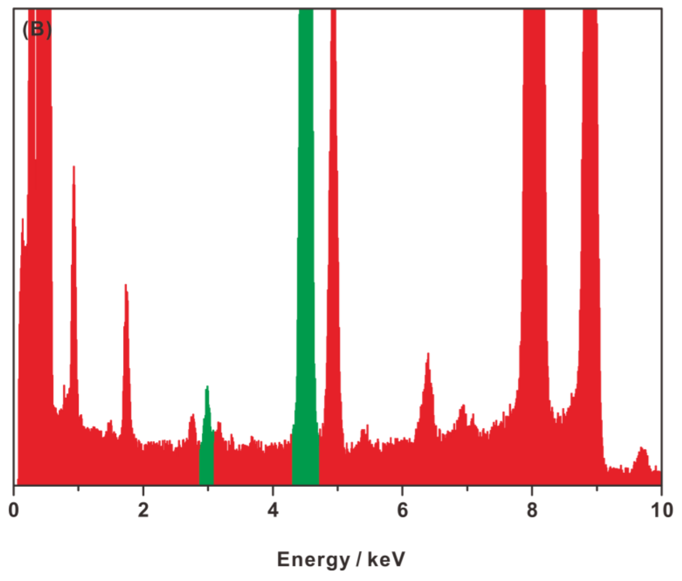

Element	Weight %	Atomic %
Ag L	4.68%	2.13%
Ti K	95.32%	97.87%

Figure S4. EDS spectra of (A) 2.5%Ag/$TiO_{2\ \{001\}}$ and (B) 2.1%Ag/$TiO_{2\ \{101\}}$.

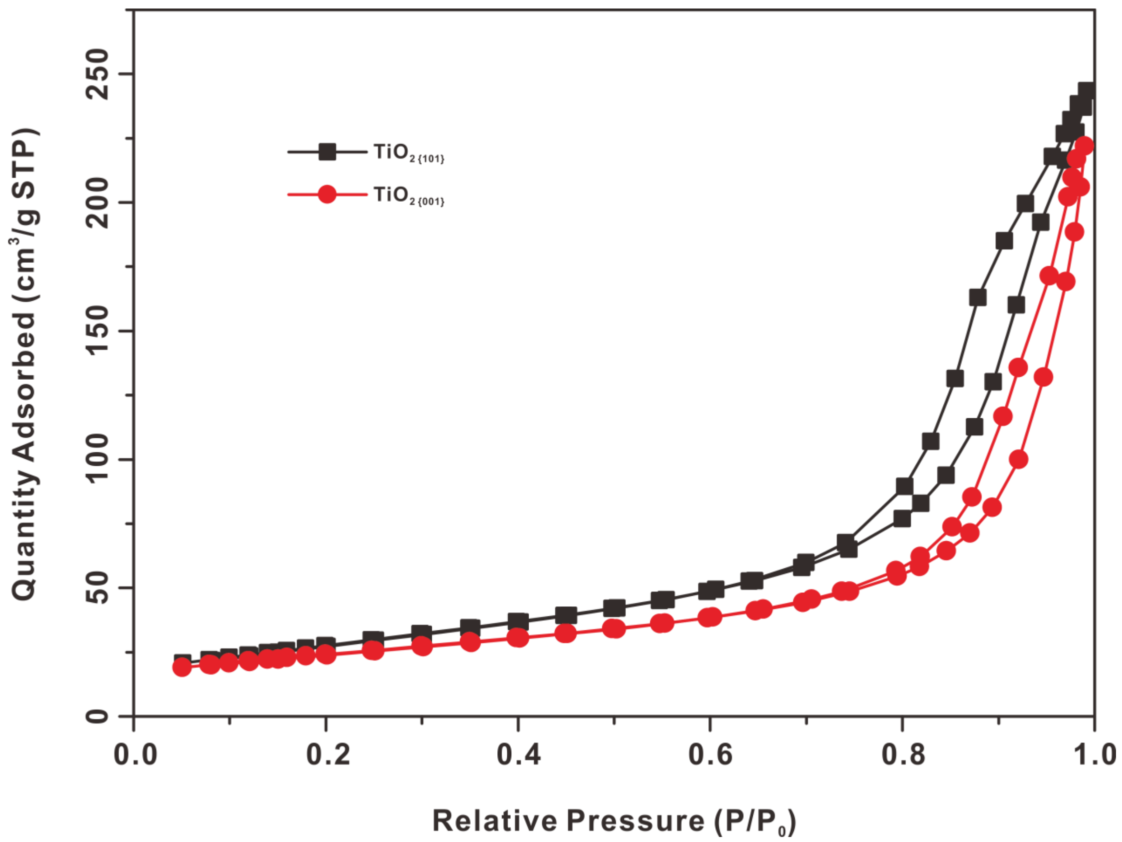

Figure S5. N_2 adsorption and desorption curves of $TiO_{2\ \{101\}}$ and $TiO_{2\ \{001\}}$ samples.

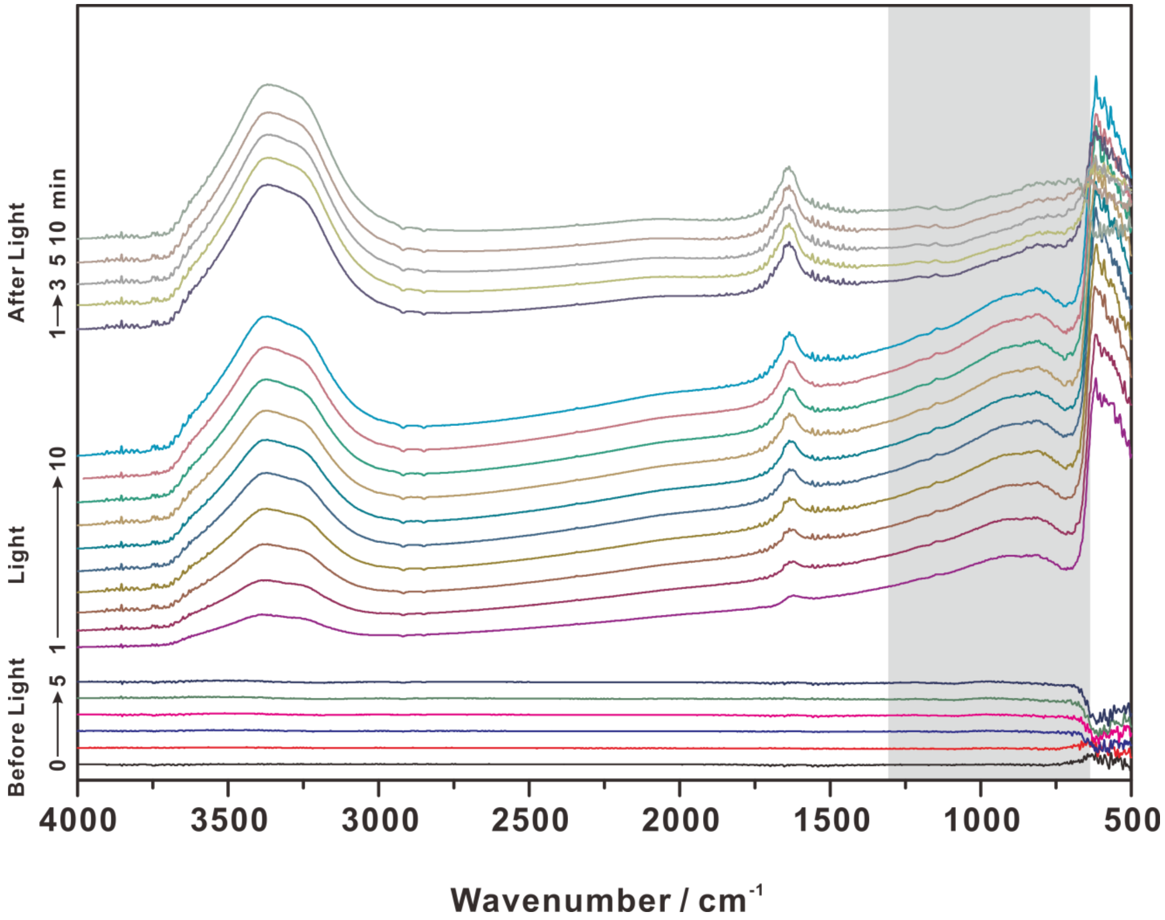

Figure S6. Operando ATR-FTIR of the aqueous phase photocatalytic reaction in the (Ar + O_2) atmosphere before, upon, and after light irradiation on 2.5%Ag/TiO_2 {001}.

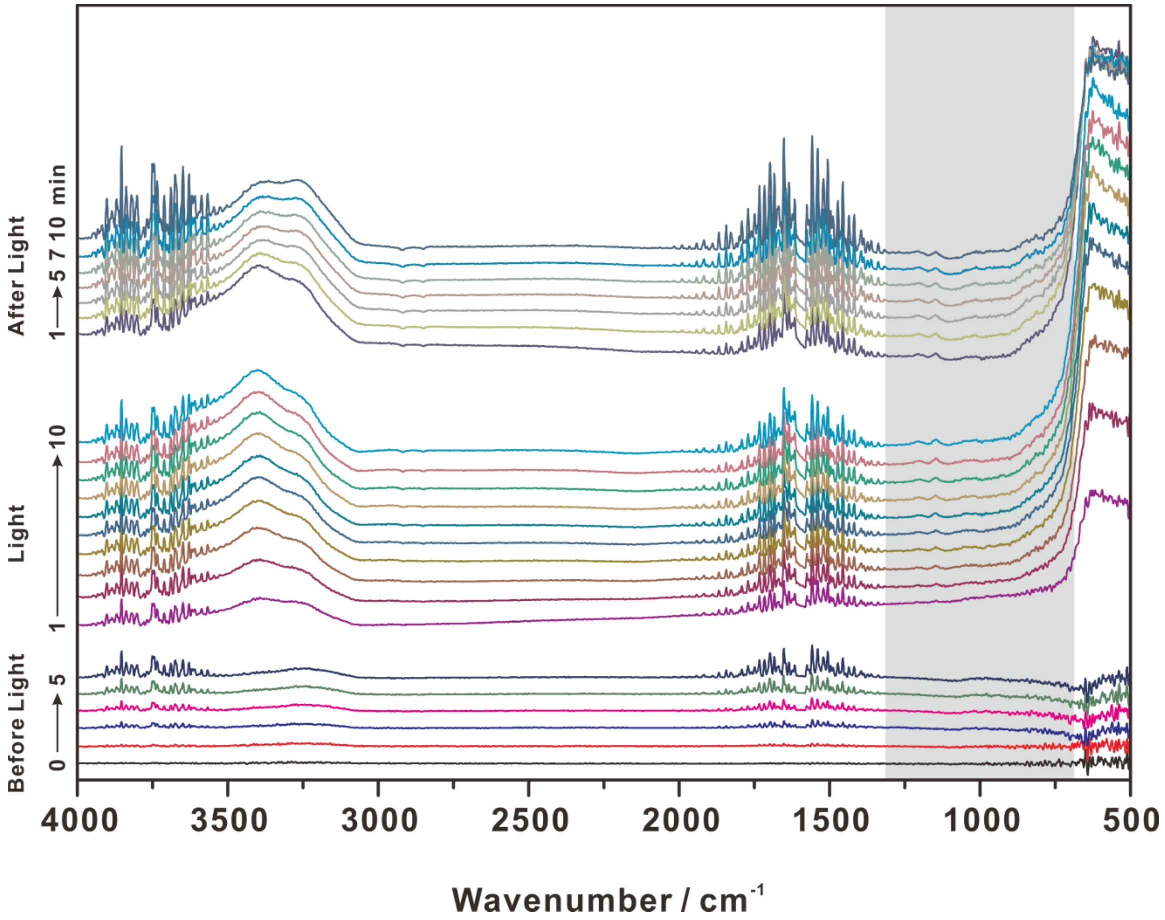

Figure S7. Operando ATR-FTIR of the aqueous phase photocatalytic reaction in the (CH_4 + O_2) atmosphere before, upon, and after light irradiation on 2.5%Ag/$TiO_{2\ \{001\}}$.

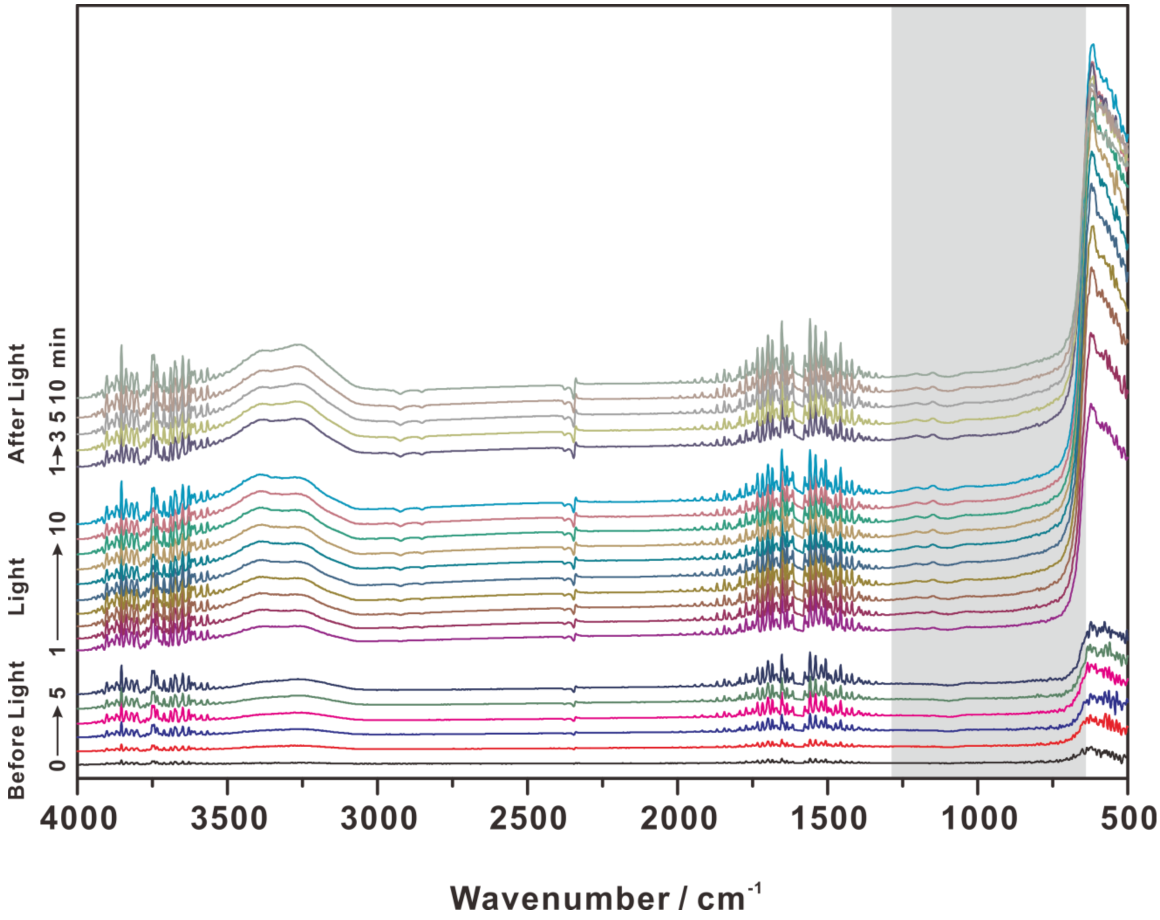

Figure S8. Operando ATR-FTIR of the aqueous phase photocatalytic reaction in the (Ar + O_2) atmosphere before, upon, and after light irradiation on 2.1%Ag/TiO_2 {101}.

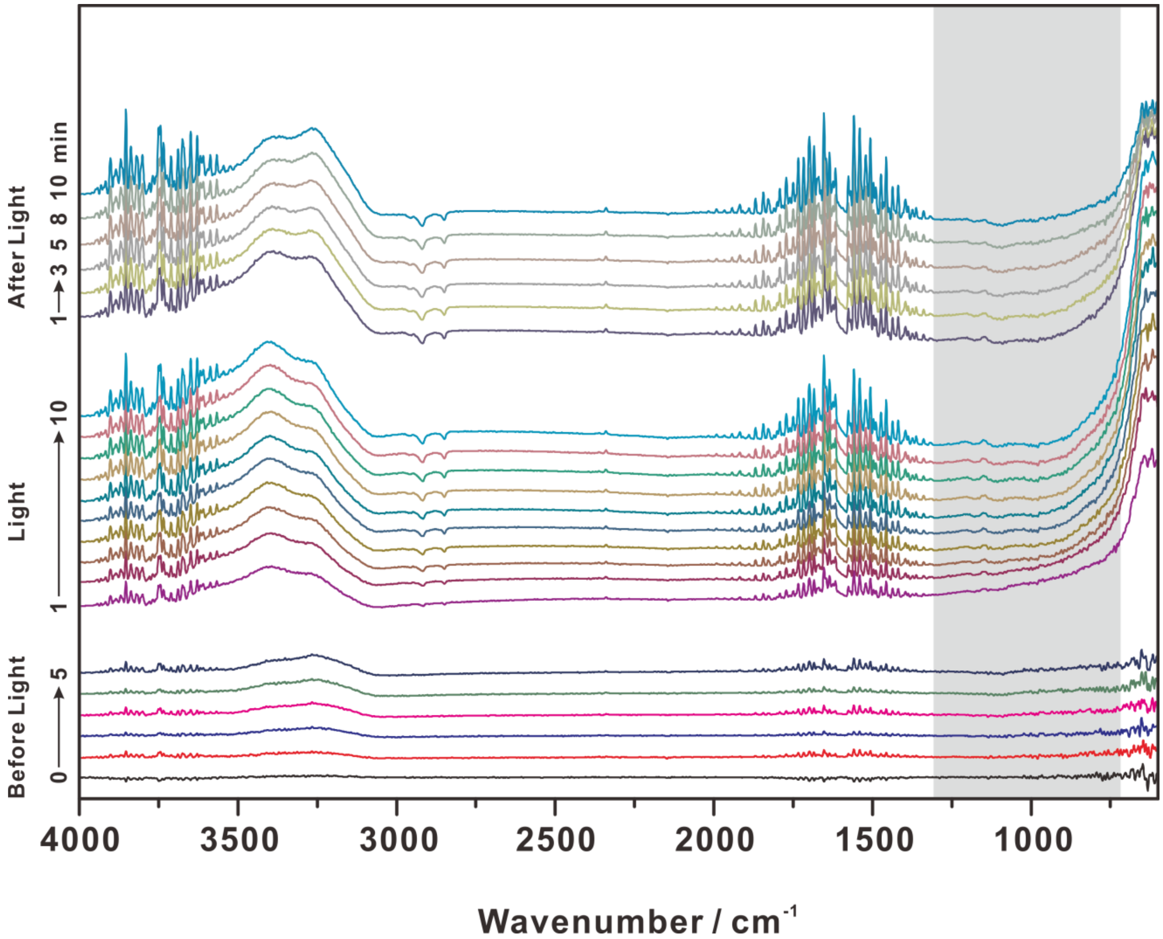

Figure S9. Operando ATR-FTIR of the aqueous phase photocatalytic reaction in the (CH_4 + O_2) atmosphere before, upon, and after light irradiation on 2.1%Ag/$TiO_{2\ \{101\}}$.

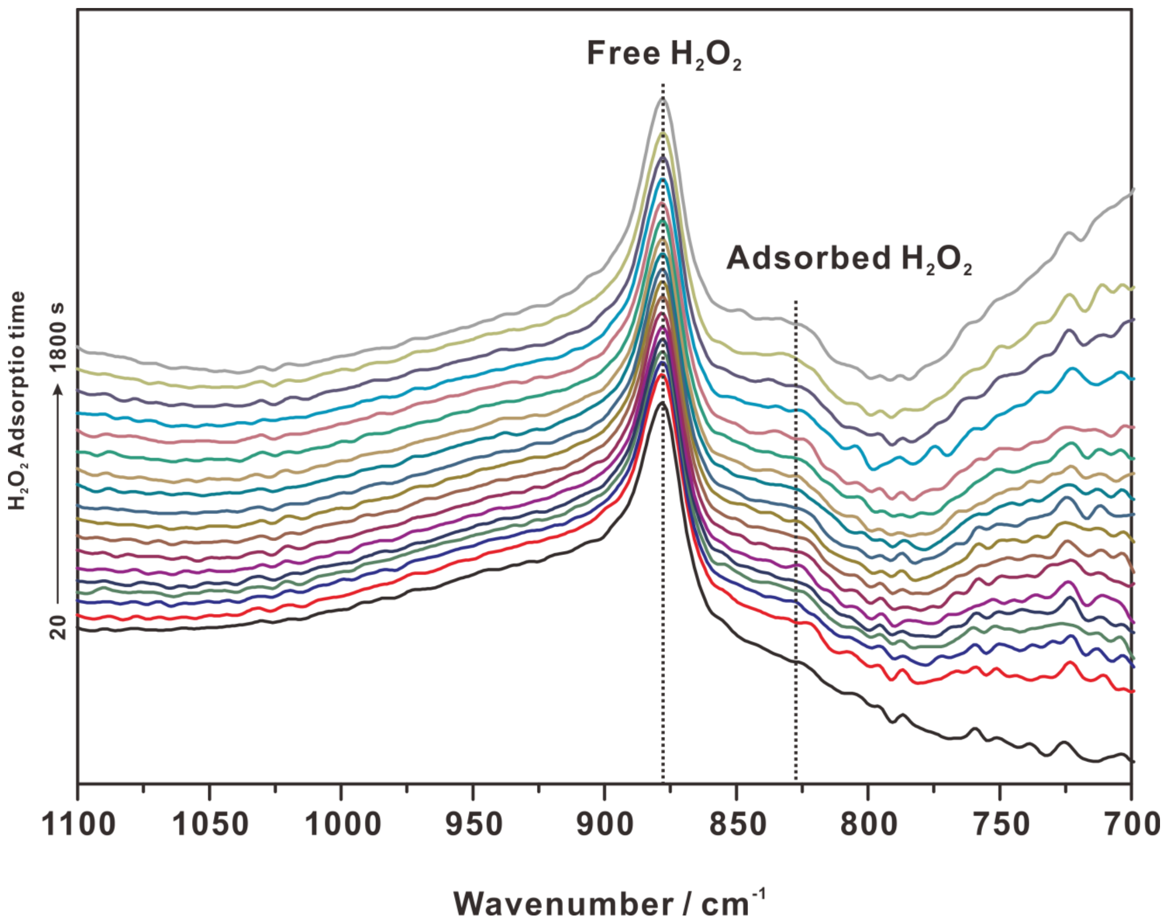

Figure S10. Operando FTIR of the adsorption of H_2O_2 on the surface of $TiO_{2\ \{001\}}$.

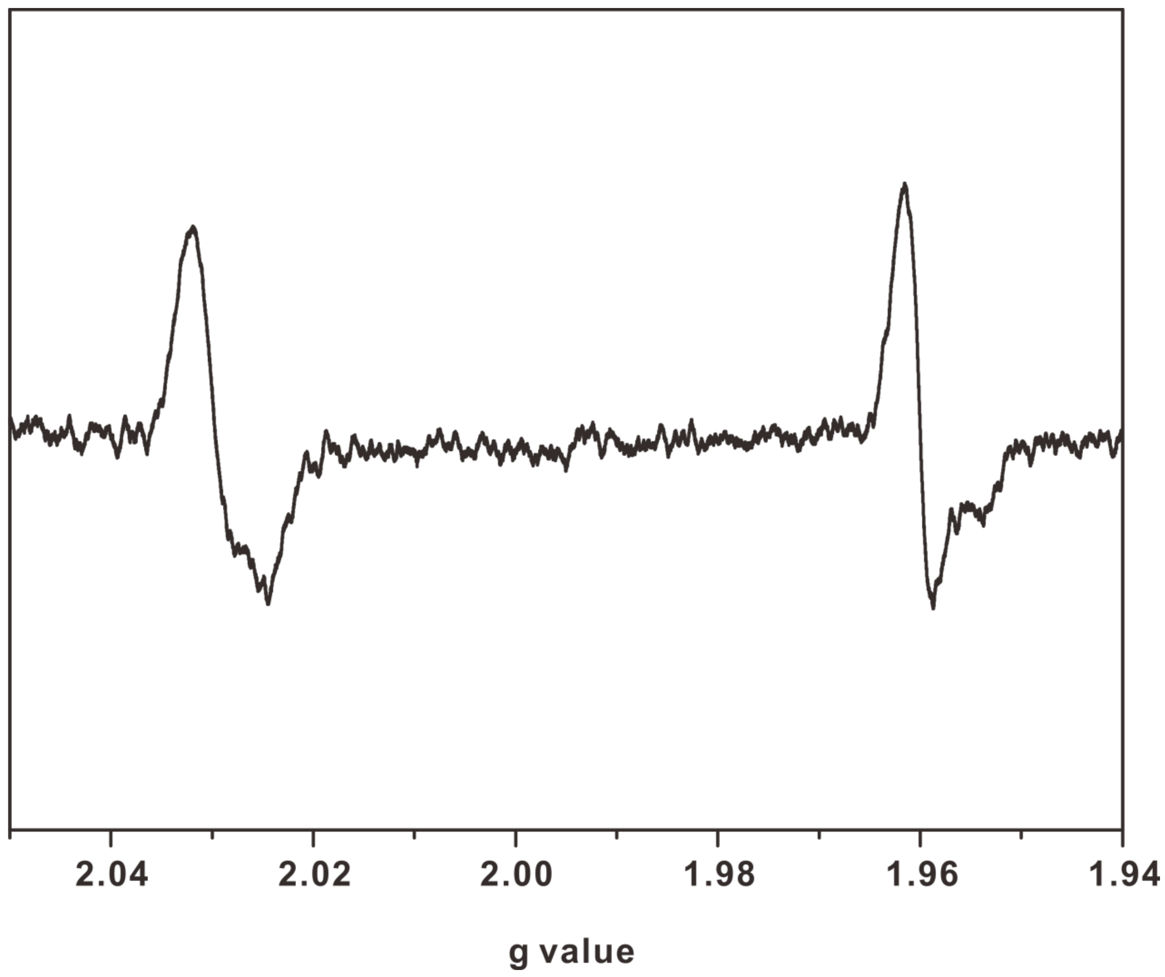

Figure S11. ESR of the empty in situ ESR tube.

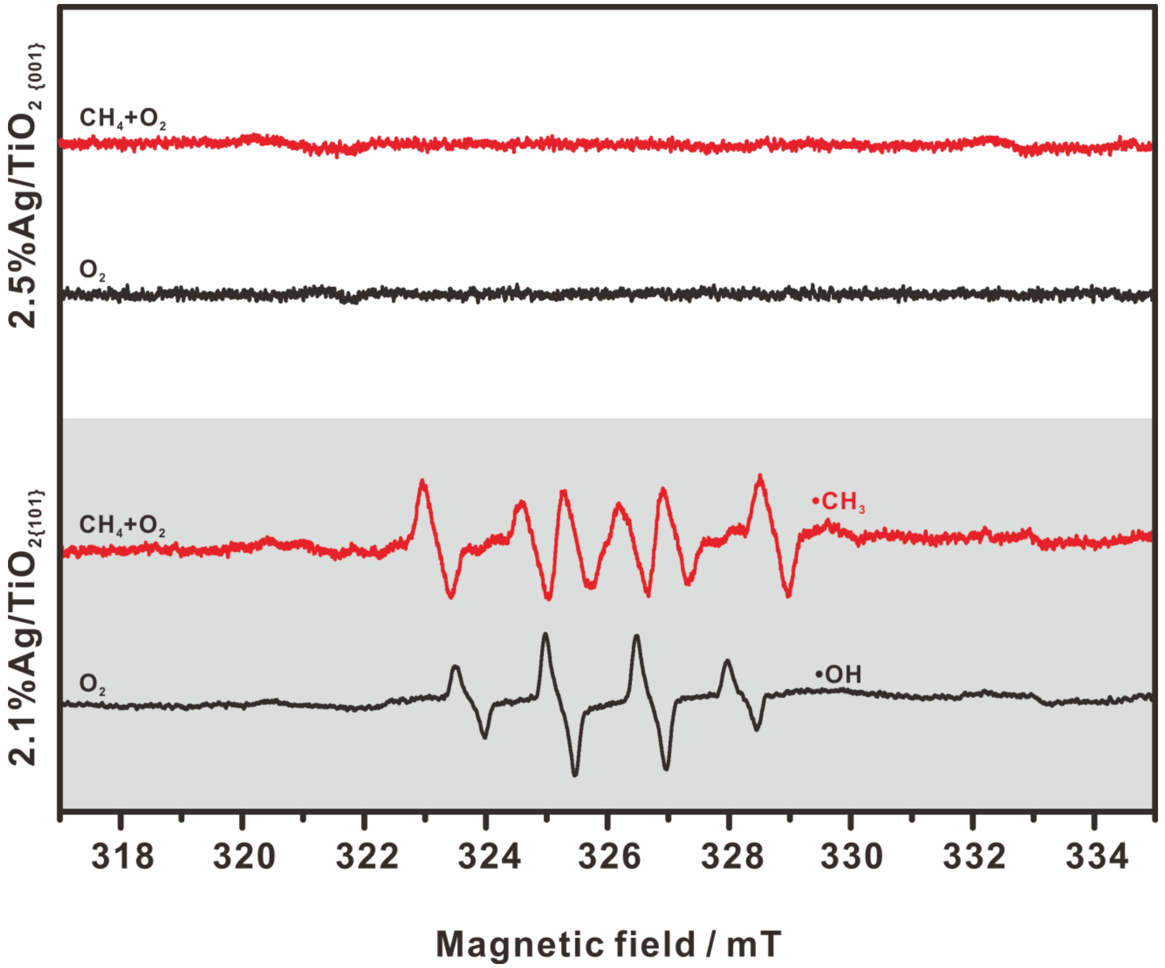

Figure S12. ESR spectra of Ag/TiO_2 in aqueous solution with O_2 and CH_4 + O_2 dissolved under light irradiation for 10 min. DMPO was added to the reaction mixture as the radical trapping agent. For 2.1%$Ag/TiO_{2\,\{101\}}$ in aqueous solution with O_2 dissolved, the signals with intensity ratio of 1:2:2:1 ($2a_N + a_H$ = 45 Guass) can be assigned to •OH trapped by DMPO. When the CH_4 was introduced into the the O_2−$Ag/TiO_{2\,\{001\}}$ system ((CH_4+O_2)−$Ag/TiO_{2\,\{001\}}$), the signal of •OH decrease obviously, and a new signal ($2a_N + a_H$ = 56 Guass) corresponding to •CH_3 occurs. However, for 2.5%$Ag/TiO_{2\,\{001\}}$ in aqueous solution with O_2 and (CH_4 + O_2) dissolved, there are almost no •CH_3 and •OH generated in the photocatalytic CH_4 oxidation. The a_N and a_H are the hyperfine coupling constants of the ^{14}N and ^{1}H atoms.

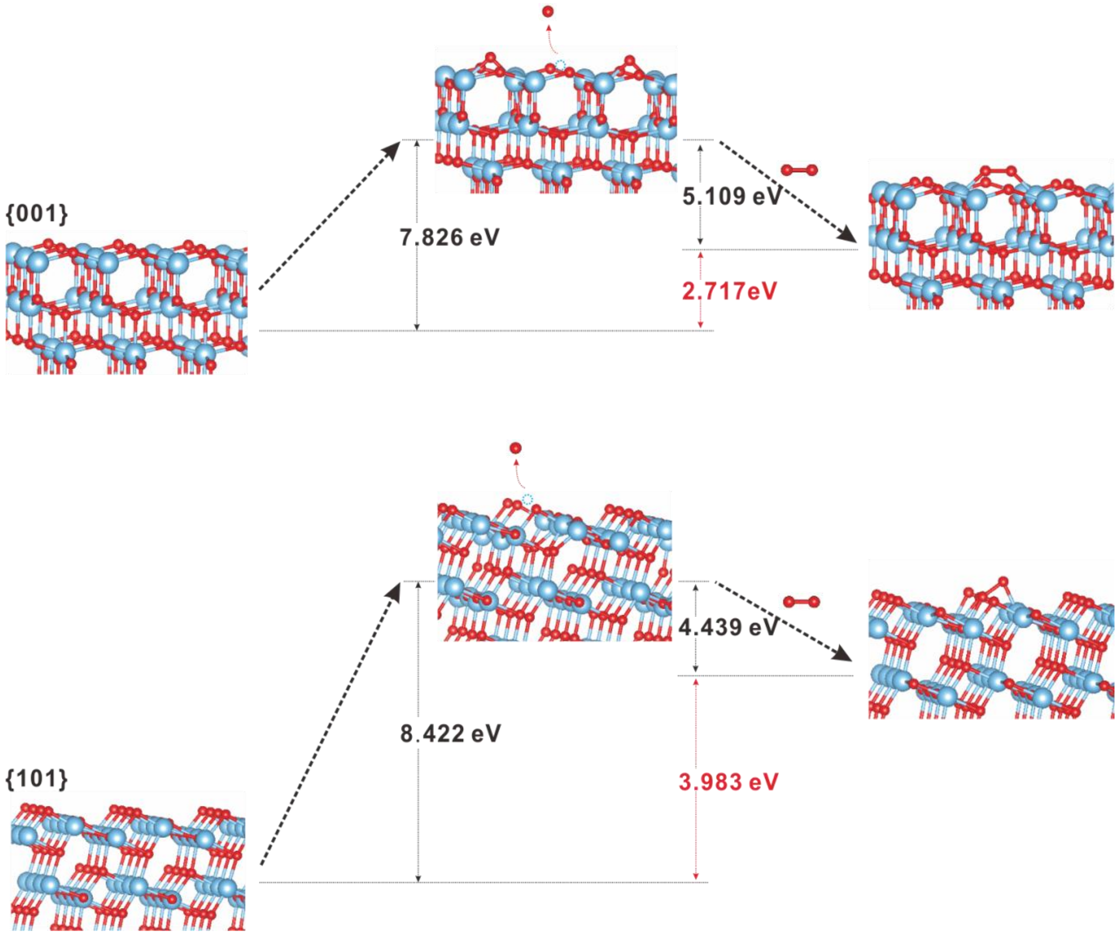

Figure S13. Theoretical calculation for the formation of oxygen vacancy and the adsorption of O_2 on {001} and {101} facets of TiO_2. Calculations based on Density functional theory (DFT) were performed with Vienna Ab initio Simulation Package (VASP). Generalized gradient approximation (GGA) by Perdew-Burke-Ernzerhof (PBE) functional and all-electron projector augmented wave (PAW) method are employed. An energy cutoff of 450 eV for the plane wave basis set was adopted. The{001} and {101} facets of anatase-TiO_2 were modeled by (3×3) and (2×2) supercell, respectively. O vacancy was modeled by removing a surface O from the supercells. Red ball represents oxygen atom, and blue ball represents Titanium atom.

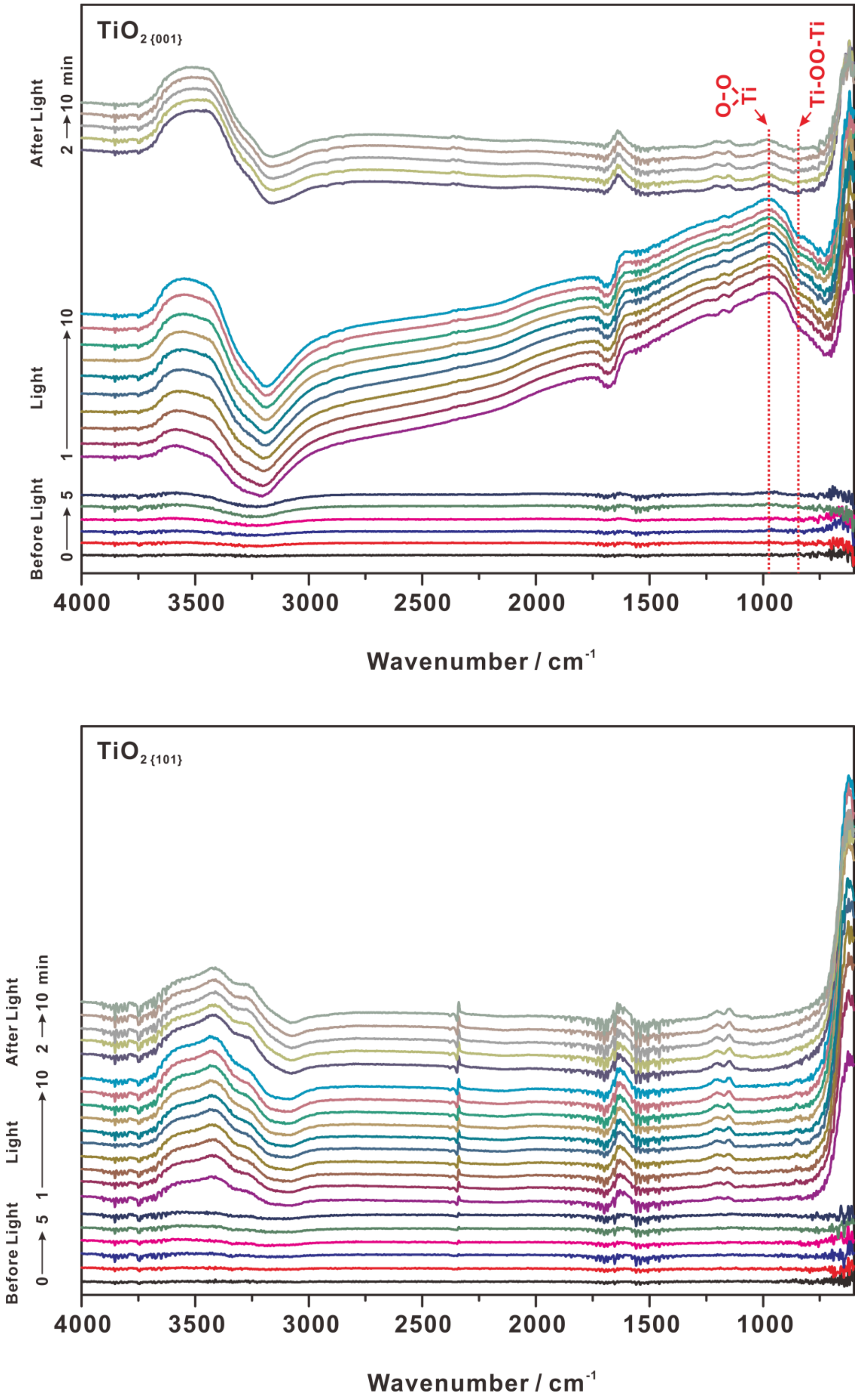

Figure S14. Operando ATR-FTIR of the aqueous phase photocatalytic reaction in the (Ar + O_2) atmosphere before, upon, and after light irradiation on $TiO_{2\ \{001\}}$ and $TiO_{2\ \{101\}}$.

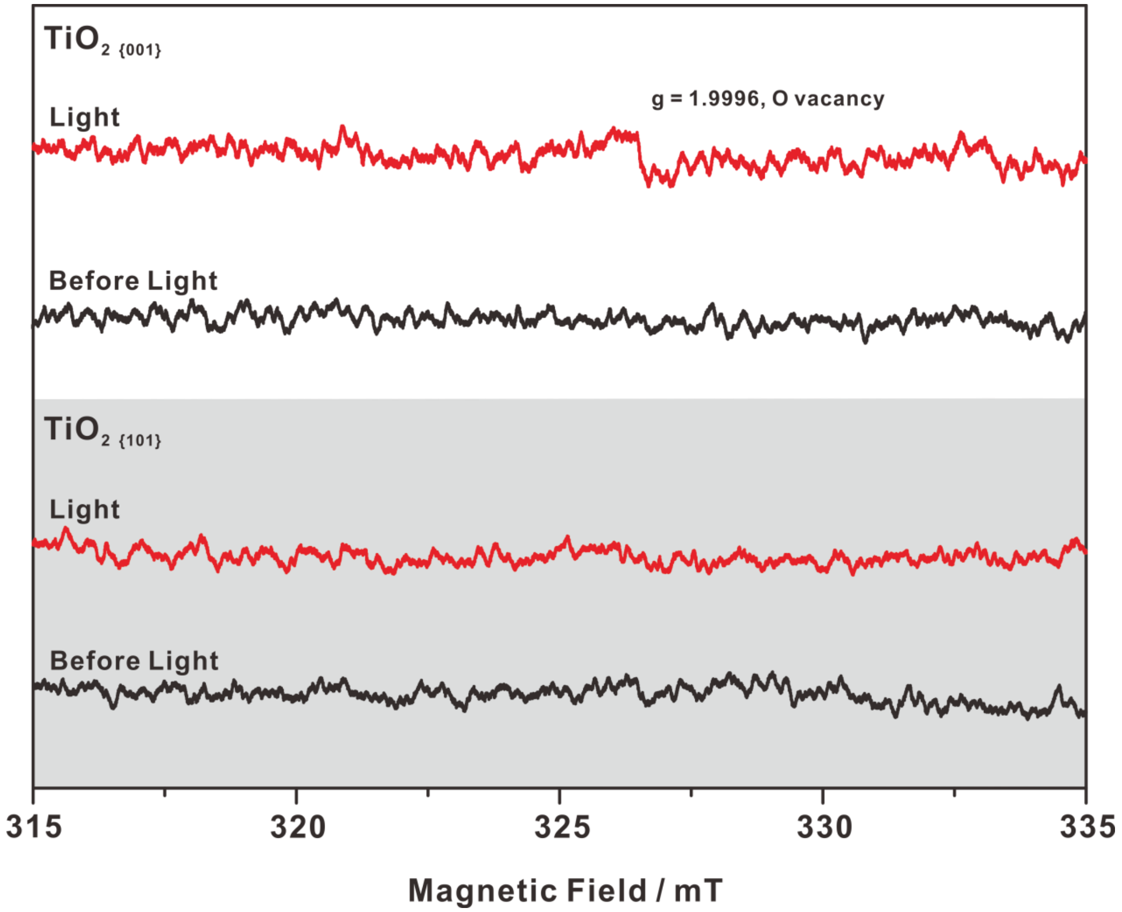

Figure S15. In situ ESR spectra for $TiO_{2\ \{001\}}$ and $TiO_{2\ \{101\}}$ with 300 μmol H_2O loading in O_2 atmospheres before and during light irradiation.

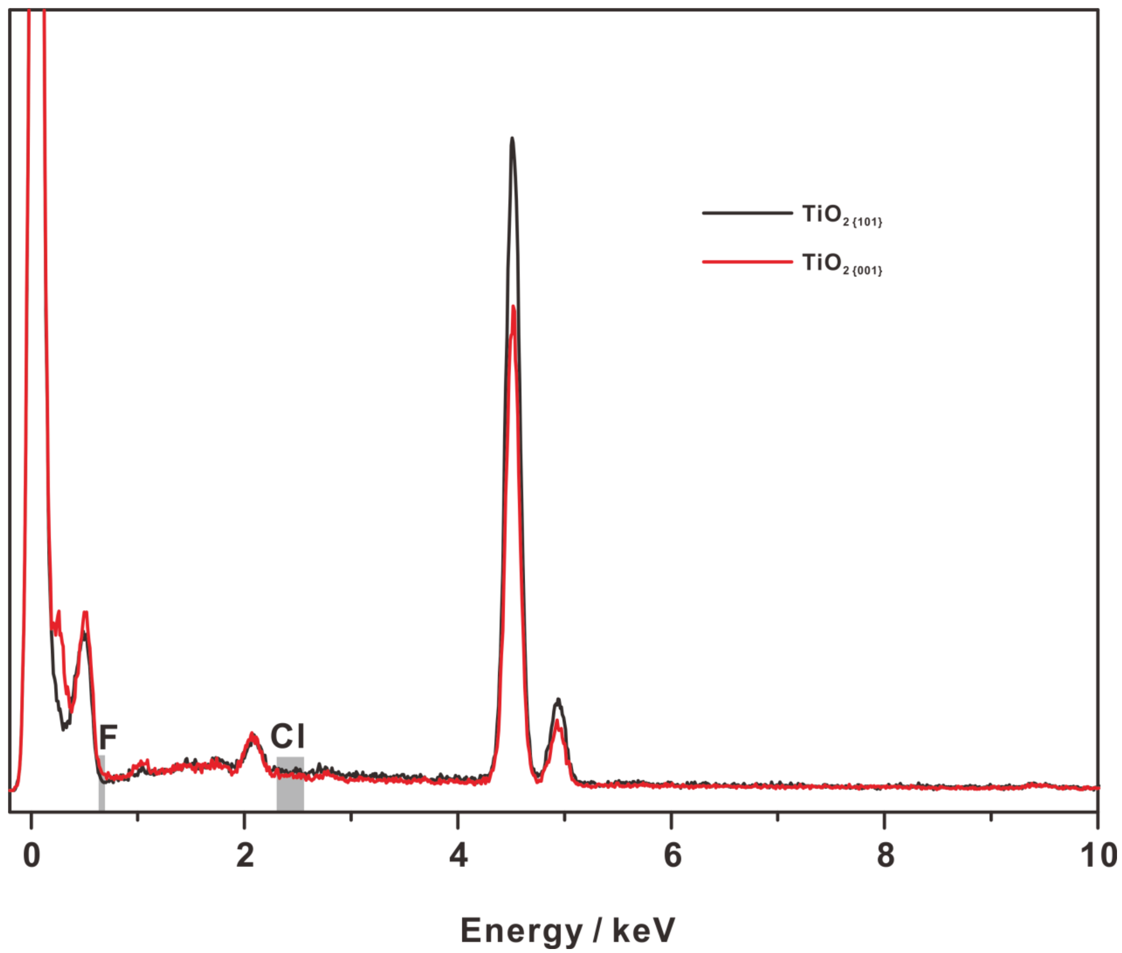

Figure S16. EDX spectra of $TiO_{2\ \{101\}}$ and $TiO_{2\ \{001\}}$.

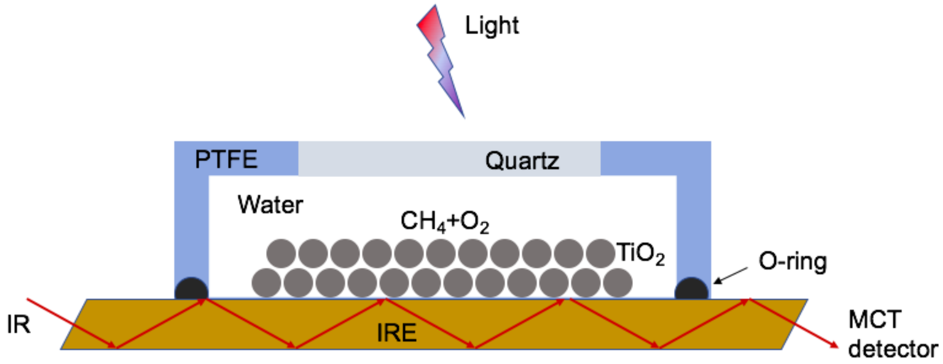

Figure S17. The schematic diagram of in-situ attenuated total reflectance Fourier transform infrared spectroscopy (in-situ ATR-FTIR) with aqueous phase in a home-made spectral cell, created by a co-author (Huiwen Lin).

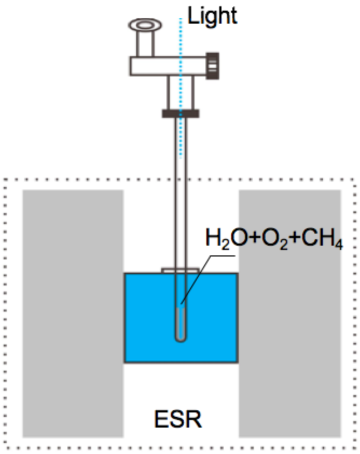

Figure S18. The schematic diagram of in-situ electron spin-resonance spectroscopy (in-situ ESR) in a home-made spectral cell, created by the first author (Ningdong Feng).

Reference

1 Xie, J. *et al.* Highly selective oxidation of methane to methanol at ambient conditions by titanium dioxide-supported iron species. *Nat. Catal* **1**, 889-896, doi:10.1038/s41929-018-0170-x (2018).

2 Song, H. *et al.* Selective Photo-oxidation of Methane to Methanol with Oxygen over Dual-Cocatalyst-Modified Titanium Dioxide. *ACS Catal.* **10**, 14318-14326, doi:10.1021/acscatal.0c04329 (2020).

3 Song, H. *et al.* Direct and Selective Photocatalytic Oxidation of CH4 to Oxygenates with O2 on Cocatalysts/ZnO at Room Temperature in Water. *J. Am. Chem. Soc.* **141**, 20507-20515, doi:10.1021/jacs.9b11440 (2019).

4 Murcia-López, S., Villa, K., Andreu, T. & Morante, J. R. Partial Oxidation of Methane to Methanol Using Bismuth-Based Photocatalysts. *ACS Catal.* **4**, 3013-3019, doi:10.1021/cs500821r (2014).

5 Villa, K., Murcia-López, S., Andreu, T. & Morante, J. R. Mesoporous WO3 photocatalyst for the partial oxidation of methane to methanol using electron scavengers. *Appl. Catal. B* **163**, 150-155, doi:https://doi.org/10.1016/j.apcatb.2014.07.055 (2015).

6 Villa, K., Murcia-López, S., Morante, J. R. & Andreu, T. An insight on the role of La in mesoporous WO3 for the photocatalytic conversion of methane into methanol. *Appl. Catal. B* **187**, 30-36, doi:https://doi.org/10.1016/j.apcatb.2016.01.032 (2016).
